# Genome-wide identification and comprehensive analyses of *NAC* transcription factor gene family and expression analysis under *Fusarium kyushuense* and drought stress conditions in *Passiflora edulis*

**DOI:** 10.3389/fpls.2022.972734

**Published:** 2022-08-25

**Authors:** Qiang Yang, Binqi Li, Hafiz Muhammad Rizwan, Kaiwei Sun, Jiajing Zeng, Meng Shi, Tianxin Guo, Faxing Chen

**Affiliations:** College of Horticulture, Fujian Agriculture and Forestry University, Fuzhou, China

**Keywords:** passion fruit, synteny analysis, multicollinearity, micro-RNA, gene expressions

## Abstract

The *NAC* gene family is one of the largest plant transcription factors (TFs) families and plays important roles in plant growth, development, metabolism, and biotic and abiotic stresses. However, *NAC* gene family has not been reported in passion fruit (*Passiflora edulis*). In this study, a total of 105 *NAC* genes were identified in the passion fruit genome and were unevenly distributed across all nine-passion fruit chromomere, with a maximum of 48 *PeNAC* genes on chromosome one. The physicochemical features of all 105 *PeNAC* genes varied including 120 to 3,052 amino acids, 3 to 8 conserved motifs, and 1 to 3 introns. The *PeNAC* genes were named (*PeNAC001–PeNAC105*) according to their chromosomal locations and phylogenetically grouped into 15 clades (NAC-a to NAC-o). Most PeNAC proteins were predicted to be localized in the nucleus. The *cis*-element analysis indicated the possible roles of *PeNAC* genes in plant growth, development, light, hormones, and stress responsiveness. Moreover, the *PeNAC* gene duplications including tandem (11 gene pairs) and segmental (12 gene pairs) were identified and subjected to purifying selection. All PeNAC proteins exhibited similar 3D structures, and a protein–protein interaction network analysis with known *Arabidopsis* proteins was predicted. Furthermore, 17 putative ped-miRNAs were identified to target 25 *PeNAC* genes. Potential TFs including *ERF*, *BBR-BPC*, *Dof*, and *bZIP* were identified in promoter region of all 105 *PeNAC* genes and visualized in a TF regulatory network. GO and KEGG annotation analysis exposed that *PeNAC* genes were related to different biological, molecular, and cellular terms. The qRT-PCR expression analysis discovered that most of the *PeNAC* genes including *PeNAC001, PeNAC003, PeNAC008, PeNAC028, PeNAC033, PeNAC058, PeNAC063*, and *PeNAC077* were significantly upregulated under *Fusarium kyushuense* and drought stress conditions compared to controls. In conclusion, these findings lay the foundation for further functional studies of *PeNAC* genes to facilitate the genetic improvement of plants to stress resistance.

## Introduction

Gene expression regulation plays an important role in various biological and physiological functions. Transcription factors (TFs) are fundamental regulatory elements at the transcriptional level and play an important role in protein evolution and crop improvement (Martínez-Ainsworth and Tenaillon, 2016). TFs modulate *cis*-acting elements of different signaling pathways by combination thereby stimulating, or repressing the expression of target genes (Hernández and Sanan-Mishra, 2017; Pascual et al., 2018). The TF typically contains four functional domains including a DNA binding region, an oligomerization site, a transcription regulation domain, and a nuclear localization (Du et al., 2012). Based on previous research on *Arabidopsis thaliana* TF families, a total of 2296 TFs were included in the PlantTFDB database and were classified into 58 families (Jin et al., 2014; Munir et al., 2020). As a class of TFs with a variety of biological functions, NAC TFs are unique to plants and belong to one of the largest TF families, and widely exist in eukaryotes (Duval et al., 2002). The NAC TF family is named after three very important proteins with analogous DNA-binding domains including no apical meristem (NAM) from *Petunia hybrida*, the *Arabidopsis* transcription activation factors (ATAF1 and ATAF2), and cup-shaped cotyledon 2 (CUC2), that formed a conserved NAC domain (Souer et al., 1996; Aida et al., 1997). NAM domains are associated with plant growth and development (Jensen et al., 2008; Trupkin et al., 2019), while ATAF1/2 and ATCUC are tangled in stress, defense response elements, and embryonic, floral, and apical meristem development, respectively (Lu et al., 2007; Tran et al., 2009; Wang et al., 2009).

Protein structure analysis revealed that the NAC TF family is a novel type and N-terminal encodes a highly conserved DNA-binding domain of about 160 amino acids, which facilitates the binding of target genes to N-terminal *cis*-elements, as well as different transactivation domains at the C-terminus (Olsen et al., 2005). The NAC domain is further classified into five subdomains including A to E and is related to different functions such as nuclear localization and DNA-binding of homodimers or heterodimers (Khedia et al., 2018). The F to O NAC subdomains are not classified according to conserved motifs and are called NAC-like proteins (Tran et al., 2009). The NAC subdomains such as A, C, and D are conserved at N-terminal and contain nuclear localization signals, while the subdomains including B and E are diverse and might be responsible for different functions (Ooka et al., 2003). The C-terminus exhibits the same highly diverse amino acid sequence, rich in serine, threonine, proline, glutamic acid, amino acid residues, etc., which plays a significant role in transcriptional regulation (Kim et al., 2006).

The NAC TF family members play a significant role in the regulation of plant growth, metabolism and development (Liu et al., 2020), cell wall biosynthesis (Hussey et al., 2011; Zhang et al., 2020), hormone signal transduction (Moschen et al., 2019; Liu et al., 2022), leaf senescence (Kim et al., 2016), fruit ripening (Jing et al., 2018; Zhang et al., 2018), seed development (Han et al., 2014), the stress response of plants to biotic and abiotic stresses (Shao et al., 2015; Raza, 2021), flower morphogenesis (Hendelman et al., 2013), and wood formation (Ohtani et al., 2011). The overexpression of *ANAC019*, *ANAC055*, and *ANAC072* enhanced drought, cold and salt tolerance in *Arabidopsis* (Tran et al., 2004). Furthermore, the overexpression of *SNAC1* (Saad et al., 2013), *ONAC22* (Hong et al., 2016), *ONAC045* (Zheng et al., 2009), and *OsNAC52* (Gao et al., 2010) enhanced tolerance to abiotic stresses in rice (*Oryza sativa* L.). Plants activate the defense response genes through disease resistance pathways such as ethylene (ET), jasmonic acid (JA), and salicylic acid (SA) to activate the plant disease resistance defense response (Yang et al., 2019). For example, overexpression of the *StNAC* gene in potato (*Solanum tuberosum*) improved the *Phytophthora infestans* infection and wounding treatment (Collinge and Boller, 2001). Overexpression of *HvNAC6* gene barley (*Hordeum vulgare*) induced resistance toward *Blumeria graminis* contamination (Jensen et al., 2007). In rice, *OsNAC4* responds to the virulent bacterial strain of *Acidovorax avenae* N1141 (Kaneda et al., 2009) and ATAF1 regulates the defense response in contradiction of various pathogens (Wang et al., 2009).

Furthermore, micro-RNAs (miRNAs) are approximately 21–30 nucleotides in length and are single-stranded non-coding RNAs that play important roles in cellular mechanisms, conferring stress resistance through translational repression or cleavage of transcriptional or post-transcriptional (Kidner and Martienssen, 2005; O’Brien et al., 2018). Asefpour Vakilian (2020) reported that the miRNA plays a significant role under plant stresses. In recent years, with the development of bioinformatics, molecular biology techniques and the crucial role of *NAC* genes, the gnome wide identification of *NAC* genes has already been reported in numerous plants including 117 *NAC* genes in *Arabidopsis* and 151 in rice (Ooka et al., 2003), 87 genes in maize (Wang G. et al., 2020), 145 genes in sorghum (*Sorghum bicolor*) (Kadier et al., 2017), 132 genes in peanut (*Arachis hypogaea*) (Li P. et al., 2021), 113 genes in *Medicago sativa* (Min et al., 2020), 90 genes in eggplant (*Solanum melongena*) (Wan et al., 2021), 102 genes in apricot (*Prunus sibirica*) (Xu et al., 2021), and cacao (*Theobroma cacao*) (Shen et al., 2019), 167 genes in barley (Murozuka et al., 2018), 64 genes in pitaya (*Hylocereus*) (Hu et al., 2022), 114 genes in walnut (*Juglans mandshurica*) (Li X. et al., 2021), 114 genes in longan (*Dimocarpus longan*) (Munir et al., 2020), 154 genes in tobacco (*Nicotiana tabacum*) (Li et al., 2018), 91 genes in cucumber (*Cucumis sativus*) (Liu et al., 2018), 180 genes in apple (*Malus domestica*) (Su et al., 2013), 147 genes in foxtail millet (*Setaria italica*) (Puranik et al., 2013), 55 genes in jujuba (*Ziziphus jujuba*) (Li M. et al., 2021), 163 genes in poplar (*Populus Trichocarpa*) (Hu et al., 2010), 152 genes in soyabean (*Glycine max*) (Le et al., 2011), and 104 genes in pepper (*Capsicum annuum*) (Diao et al., 2018), respectively. However, the *NAC* gene family has not been studied in passion fruit.

Passion fruit belongs to the *Passifloraceae* family and is a perennial vine, widely cultivated in subtropical and tropical regions of the world. It has important economic value and is demanded because of its fresh juice as raw material for the food and beverage industry. Passion fruit is rich in nutrients and its roots, stems, and leaves are also used in the pharmaceutical industry (Rizwan et al., 2021). Passion fruit is often affected by adverse environmental factors during growth and development, resulting in reduced yield, quality, and huge economic losses (Rizwan et al., 2021; Yan C. et al., 2021). The genetic improvement of desirable traits in plants is important, especially to increase productivity, quality, and resistance to stresses. Previous studies on *NAC* genes have shown vital roles in plant growth, development, disease and stresses resistance. The recently published passion fruit genome (Ma et al., 2021) provides a great opportunity to explore the *NAC* family members in the passion fruit genome.

In the current study, a comprehensive genome-wide identification and analysis of the *NAC* family members were performed in passion fruit genome. Moreover, the physicochemical characteristics, phylogenetic relationship, gene structure, chromosomal location, conserved motifs, collinearity, and gene duplication of passion fruit *NAC* genes were analyzed in detail. The subcellular localization, protein–protein interaction, and *cis*-regulatory element analyses of the passion fruit *NAC* gene were speculated. In addition, the passion fruit *NAC* gene expressions in different tissues were analyzed using the existing passion fruit RNA-seq data. Furthermore, the qRT-PCR (real-time quantitative reverse transcription PCR) expressions of selected *NAC* genes in diverse passion fruit tissues under biotic (*Fusarium kyushuense* pathogenic fungal stress) and abiotic stress (drought stress) conditions were examined to better understand the functions of passion fruit *NAC* genes. The current study results laid a foundation for understanding the regulatory mechanism of the *NAC* genes, which will be useful for further functional studies of *NAC* genes and to promote the genetic improvement of passion fruit growth, development, and resistance to stresses.

## Materials and methods

### Identification of passion fruit *NAC* genes

In order to identify the *NAC* gene family members in the passion fruit genome, the *Arabidopsis* NAC proteins were downloaded from the TAIR database (the *Arabidopsis* Information Resource^1^; accessed on 14 April 2022), *P. trichocarpa* NAC proteins from PlantTFDB version 5.0^2^ (accessed on 14 April 2022) (Tian et al., 2020), *C. sativus* NAC proteins from cucurbitgenomics database^3^ (accessed on 14 April 2022), *Z. jujuba* NAC from NCBI (National Center for Biotechnology Information^4^; accessed on 14 April 2022) and *T. cacao* (Belizian Criollo B97-61/B2) from the ensemble^5^ (accessed on 14 April 2022). The passion fruit proteins, CDS, genome and gff files were downloaded from the passion fruit genome^6^ (accessed on 14 April 2022) (Ma et al., 2021). In order to find possible members of the NAC family members in the passion fruit genome, we used two methods, by BLASTp (Basic Local Alignment Search Tool for proteins) and HMMER (Hidden Markov Models) search tool *via* TBtools software (version 1.0984735^7^) (accessed on 14 April 2022) (Chen et al., 2020), with default mode.

The NAM (PF02365) domain HMM file was downloaded from Pfam^8^ (accessed on 14 April 2022) and was used for the identification of *NAC* genes in passion fruit genome *via* a simple HMM search package in TBtools software (Chen et al., 2020). Additionally, the outcomes of the two methods were merged and the presence of the NAM domain in each *NAC* gene was confirmed by different online tools including CDD^9^ (accessed on 14 April 2022), SMART^10^ (accessed on 14 April 2022), IinterProScan^11^ (accessed on 14 April 2022).

### Physicochemical characteristics and phylogenetic analyses of *PeNAC* genes

The physicochemical properties of *PeNAC* genes including molecular weight (MW), amino acids (aa), ORF length (bp), and isoelectric point (pI) were calculated by ExPASy^12^ (accessed on 24 April 2022) (Gasteiger et al., 2005). The PeNAC proteins subcellular localization were predicted by plant-mPLoc-2^13^ (accessed on 24 April 2022) (Chou and Shen, 2010). The NAC protein sequences of *P. edulis* (PeNAC), *P. trichocarpa* (PNAC), and *A. thaliana* (AtNAC) were aligned by MEGA software (Molecular Evolutionary Genetics Analyses) version 10.1.8^14^ (accessed on 14 April 2022) (Kumar et al., 2018), and an NJ (neighbor-joining) tree was constructed using 1,000 bootstrap and all other parameters were set to default. Finally, the phylogenetic tree was visualized using the online tool iTOL (Interactive Tree of Life)^15^ (accessed on 24 April 2022).

### Gene structure and motif analysis of *PeNAC* genes

The *PeNAC* gene structures (exon-intron) were drawn by TBtools software (Chen et al., 2020). The PeNAC proteins conserved motifs were predicted by MEME web tool version 5.4.1 (Multiple Expectation Maximization for Motif Elicitation)^16^ (accessed on 24 April 2022) (Bailey et al., 2015) and the number of motifs was set to 10. The results of the PeNAC phylogenetic tree, exon-intron, gene structure, and conserved motifs were shown by TBtools software.

### *Cis*-regulatory element analysis of *PeNAC* genes

For the prediction of *cis*-regulatory elements in the *PeNAC* genes, the upstream promoter region (2,000 bp) of the *PeNAC* genes was extracted and predicted by Plant CARE^17^ (accessed on 24 April 2022). The *cis*-regulatory element figure was constructed by TBtools software. Furthermore, the numbers of *cis*-regulatory elements, sequences, and functions were concise and emphasized in phytohormones responsiveness, plant growth and development and stress-responsiveness categories.

### Synteny analysis and calculation of Ka/Ks values of *PeNAC* genes

Gene duplication including tandem or segmental or whole-genome duplication (WGD) provides better information about gene family development and evolution. Homologous *PeNAC* genes having only one intervening gene on the same passion fruit chromosome were considered tandem duplicated genes, whereas on the other chromosomes were segmental duplicated genes. The *PeNAC* gene Ka (non-synonymous)/Ks (synonymous) values, gene duplication, and synteny analysis were performed by TBtools software. The synteny relationships of *NAC* genes among *P. edulis*, *A. thaliana*, *T. cacao*, *C. sativus*, and *Z. jujuba* were performed and visualized using the MCScanX toolkit package of TBtools software. In addition, the multicollinearity analysis of *NAC* genes among the whole genomes of *P. edulis*, *A. thaliana*, *T. cacao*, *C. sativus*, and *Z. jujuba* whole was performed using the TBtools software package Multiple synteny Plot. The nucleotides substitution rate (Ka and Ks) and ratios (Ka/Ks) of duplicated *PeNAC* genes were calculated by TBtools software. The time of divergence (T, mya: million years ago) of *PeNAC* genes was measured using the following reference formula: T = Ks/2x (x = 6.38 10.9) (Ma et al., 2021).

### Protein–protein interaction analysis and 3D modeling of PeNAC

The online STRING database^18^ (accessed on 24 April 2022) was used to predict and generate protein--protein interaction networks between PeNAC proteins based on known *Arabidopsis* homologs. The STRING parameters were adjusted as follows; network type was set to full STRING network; the meaning of network edges was set to evidence; the minimum required interaction score was set to medium confidence parameter (0.4) and max number of interaction display was not exceed 10 interactors. Furthermore, the 3D (three-dimensionally) models of all 105 PeNAC proteins were predicted by the online Phyre2 tool with a 100% confidence level^19^ (Kelley et al., 2015).

### Prediction of putative micro-RNAs, gene ontology, and Kyoto encyclopedia of genes and genomes annotation analysis of *PeNAC* genes

The prediction of putative miRNA sites in the *PeNAC* gene was achieved by downloading the published passion fruit mature miRNAs (Paul et al., 2020), and then *PeNAC* genes CDS sequences were submitted to the online psRNATarget Server18^20^ (accessed on 24 April 2022) with default parameters. A network interaction among the *PeNAC* target genes and putative predicted miRNAs was constructed and displayed by Cytoscape version 3.91^21^ (accessed on 24 April 2022). In addition, *PeNAC* genes were subjected to GO (Gene Ontology) and KEGG (Kyoto Encyclopedia of Genes and Genomes) annotation analysis by submitting PeNAC protein sequences to eggNOG-MAPPER^22^ (accessed 24 April 2022) database and the enrichment analysis were performed by TBtools software.

### *PeNAC* genes transcription factor regulatory network analysis

The *PeNAC* genes TF prediction and regulatory network analysis were performed as described by Rizwan et al. (2022). The online tool Plant Transcriptional Regulatory Map (PTRM)^23^ (Tian et al., 2020) was used for the prediction of TFs in the upstream (1000-bp) regions of *PeNAC* genes with *p* ≤ 1e--5. The predicted TFs were visualized into a network using the Cytoscape software version 3.9^24^ (Kohl et al., 2011).

### Expression analyses of *PeNAC* genes in various tissues

The expression analysis of *PeNAC* genes was performed in different tissues of passion fruit under different conditions using the available transcriptional expression data as described by Rizwan et al. (2022). The sample details were as follows: the peel tissue samples of both cultivars including yellow (*P. edulis*. Flavicarpa cv Huangjin) and purple (*P. edulis*. Sims cv Tainong) were from the ripening stage; pulp samples were from fruitlet, green, version, and ripening stages of fruit development. Root tissue samples were from cold-tolerant (Pingtan-1, purple passion fruit) in two cultivation areas including limestone (L) and sandy dolomite (D) rocky desertification. Leaf samples were from cold-sensitive [yellow Huangjinguo (HJG)] and cold tolerant (purple Tainong-1) cultivars under chilling stress (CS) and normal temperature (NT) conditions. Since the FPKM (transcript reads per million mapped reads) expression values vary widely between different passion fruit tissues, the FPKM expression values were converted to log^2^FC (FC-fold change) and heatmaps were generated using TBtools software.

### Plant materials and stress treatments

To study the expression profiles of passion fruit *NAC* genes under biotic and abiotic stress, the material was subjected to abiotic stress (drought stress) and biotic stress (*Fusarium kyushuense* pathogenic fungus) conditions. For fungal infection sample preparation, fruits of yellow (*P. edulis.* Flavicarpa cv Huangjin) and purple (*P. edulis*. Sims cv Tainong) passion fruit cultivars were obtained from a commercial orchard situated in Fujian province (23°48035.200 N and 117°7008.100 E) China. The fruit surface disinfection and inoculation with *F. kyushuense* were performed following Rizwan et al. (2021) protocol. The peels from the infected areas were collected after 9 and 12 days of inoculation, whereas the non-treated fruits were used as controls. For sample preparation under drought stress conditions, seeds of both cultivars were grown in plastic pots containing peat moss and soil (2:1 ratio) in a greenhouse at 25 ± 2°C, light/dark (16/8-h), and 75–80% RH (relative humidity). After 1 month, the passion fruit seedlings were exposed to dehydration for 10 days and then rewatered. Root, stem, and leaves samples were collected into three biological replicates and frozen into liquid nitrogen, and stored at −80°C for subsequent uses. Control samples were from normally watered plants.

### RNA isolation and quantitative real-time-polymerase chain reaction

Total RNA was isolated from the collected samples with the help of the Tiangen mini-RNA extraction kit (Tiangen, China) following the instructions of the manufacturer. The quality and concentration of the RNA samples were assessed by Thermo Scientific NanoDrop 2000 UV-Vis Spectrophotometer (Thermo Scientific, United States). One μg of total RNA was used for cDNA (complementary DNA) synthesis using Takara PrimeScript™ RT Kit with gDNA eraser (TAKARA, China), and was diluted to 5× with deionized distilled water. Gene-specific primers were designed using the primer3plus online tool^25^ (accessed 24 April 2022) (Supplementary Table 1). The LightCycler^®^ 96 (Roche Applied Science, Penzberg, Germany) was used to perform qRT-PCR (quantitative real-time polymerase chain reaction) in a 20 μL reaction mixture, containing 10 μL of TB Green premixed enzyme solution (TAKARA, China), 1.0 μL each of the forward and reverse primers (100 μM), 1 μL cDNA, and 7 μL ddH_2_O.

The *PeNAC* genes were selected for qRT-PCR expression analysis based on their *cis*-acting elements and homologous sequences to NAC members that have been characterized in *Arabidopsis* and rice in response to biotic and drought stresses. Passion fruit genes encoding *Pe60S*, *PeTIF* (transcription initiation factor), and histone were used as reference genes for internal controls (Munhoz et al., 2015). The qRT-PCR reaction was performed under the following conditions including preincubation at 95°C for 30 s, 45 cycles at 95°C for 10 s, and 60°C for 30 s. In each reaction, three biological replicates were used and the relative gene expressions were normalized with the *Pe60S* gene and calculated using the 2^–ΔΔCT^ method (Schmittgen and Livak, 2008).

### Statistical analysis

One-way analysis of variance (ANOVA) was used to perform the statistical analysis between treated and controlled samples using the student’s *t*-test and was considered statistically significant if *p* < 0.05. The figures were constructed by GraphPad Prism version 9.0^26^ (accessed 28 April 2022).

## Results

### Identification and physicochemical properties of *PeNAC* genes

The *NAC* genes in the passion fruit genome were identified by two methods and their conserved domain (NAM) was further confirmed by CDD, SMART, and IinterProScan tools. After combining the results of the two methods and removing the redundant, repetitive, and unrecognized sequences, 105 *PeNAC* genes were identified in the passion fruit genome and named (*PeNAC1* to *PeNAC105*) according to their genomic location on the chromosomes (Table 1). All the PeNAC proteins contained the NAM domain. The physicochemical properties of *PeNAC* genes varied, for example, the encoded proteins range from 120 to 3052 amino acids with an average length of 480 (Table 1). PeNAC065 was the shortest protein-encoding 120 amino acids, while PeNAC019 was the largest protein-encoding 3052 amino acids (Table 1). Correspondingly, the PeNAC ORFs ranged from 363 bp (*PeNAC*065) to 9159 bp (*PeNAC*019) (Table 1). The predicted molecular weight of the PeNAC proteins ranges from 13.82 kDa (PeNAC044) to 347.13 kDa (PeNAC019) (Table 1). The predicted isoelectric points (pI) of PeNAC proteins ranged from 4.59 (PeNAC077) to 9.88 (PeNAC044), respectively (Table 1). In addition, the results of protein subcellular localization prediction showed that, except for PeNAC086 located in the chloroplast, 104 of the 105 PeNAC proteins were localized in the nucleus (Table 1).

**TABLE 1:** Physicochemical properties of *PeNAC* genes.

Gene name	Gene ID	Cr*	Location	ORF (bp)*	aa*	pI*	MW (Kda)*	GRAVY*	Domain	SCL*
*PeNAC*001	ZX.01G0001370	1	3416918:3419010+	1074	357	6.43	41.90	−0.70	NAM	Nu*
*PeNAC*002	ZX.01G0003180	1	11176273:11178627−	1080	359	5.23	41.42	−0.76	NAM	Nu
*PeNAC*003	ZX.01G0004270	1	13274035:13276995−	882	293	7.03	33.19	−0.55	NAM	Nu
*PeNAC*004	ZX.01G0007340	1	16824379:16826435−	1011	336	6.03	38.19	−0.63	NAM	Nu
*PeNAC*005	ZX.01G0007730	1	16996283:16998065+	1287	428	5.95	48.20	−0.58	NAM	Nu
*PeNAC*006	ZX.01G0008770	1	17586354:17587834−	744	247	9.34	28.27	−0.62	NAM	Nu
*PeNAC*007	ZX.01G0009760	1	17998438:18010077−	2970	989	5.52	112.73	−0.95	NAM	Nu
*PeNAC*008	ZX.01G0010000	1	18120368:18132026−	3081	1026	5.44	116.60	−0.86	NAM	Nu
*PeNAC*009	ZX.01G0011990	1	19128123:19130144+	951	316	4.63	47.45	−0.38	NAM	Nu
*PeNAC*010	ZX.01G0012360	1	19289302:19298535+	4497	1498	6.98	16.66	−0.47	NAM	Nu
*PeNAC*011	ZX.01G0013370	1	20949220:20954521+	951	316	5	35.86	−0.68	NAM	Nu
*PeNAC*012	ZX.01G0013390	1	20971684:20973747+	906	301	5.25	34.01	−0.61	NAM	Nu
*PeNAC*013	ZX.01G0013410	1	20997183:20999288+	885	294	5.16	33.13	−0.64	NAM	Nu
*PeNAC*014	ZX.01G0026100	1	32219081:32221528+	909	302	6.61	34.06	−0.57	NAM	Nu
*PeNAC*015	ZX.01G0029470	1	35556228:35558581+	1197	398	5.43	44.93	−0.46	NAM	Nu
*PeNAC*016	ZX.01G0031480	1	36811204:36812772+	1011	336	5.97	38.25	−0.48	NAM	Nu
*PeNAC*017	ZX.01G0035500	1	39090156:39111053+	8133	2711	5.65	307.41	−0.69	NAM	Nu
*PeNAC*018	ZX.01G0035710	1	39209291:39210620+	1146	381	5.88	43.17	−0.72	NAM	Nu
*PeNAC*019	ZX.01G0035750	1	39228149:39251186−	9159	3052	6.03	347.13	−0.59	NAM	Nu
*PeNAC*020	ZX.01G0040370	1	42310269:42313180+	984	327	8.97	36.46	−0.62	NAM	Nu
*PeNAC*021	ZX.01G0043020	1	43760720:43762061+	570	189	5.06	21.80	−0.65	NAM	Nu
*PeNAC*022	ZX.01G0044050	1	44304680:44307168−	1356	451	8.33	51.17	−0.78	NAM	Nu
*PeNAC*023	ZX.01G0046390	1	45621667:45622594+	534	177	5.03	21.01	−0.87	NAM	Nu
*PeNAC*024	ZX.01G0047580	1	46286914:46289756−	1170	389	7.78	44.12	−0.81	NAM	Nu
*PeNAC*025	ZX.01G0048910	1	47997171:48000479+	2007	668	5.38	76.08	−0.59	NAM	Nu
*PeNAC*026	ZX.01G0049810	1	48832502:48844661+	3587	1199	8.68	135.00	−0.66	NAM	Nu
*PeNAC*027	ZX.01G0049870	1	48987801:48989056−	771	256	6.22	29.13	−0.57	NAM	Nu
*PeNAC*028	ZX.01G0052630	1	52213982:52214731−	375	124	9.04	14.29	−0.76	NAM	Nu
*PeNAC*029	ZX.01G0056180	1	55437586:55438893+	963	321	6.13	36.76	−0.65	NAM	Nu
*PeNAC*030	ZX.01G0059700	1	57881442:57882904−	1050	349	6.27	40.02	−0.51	NAM	Nu
*PeNAC*031	ZX.01G0064530	1	62302064:62302758−	390	129	5.92	14.72	−0.65	NAM	Nu
*PeNAC*032	ZX.01G0066650	1	64155998:64158725+	678	225	5	25.74	−0.71	NAM	Nu
*PeNAC*033	ZX.01G0069360	1	67598174:67599505+	1158	386	9.08	43.93	−0.56	NAM	Nu
*PeNAC*034	ZX.01G0069750	1	68646137:68648398−	1065	354	7.67	40.09	−0.56	NAM	Nu
*PeNAC*035	ZX.01G0071590	1	72752260:72753273+	742	246	8.69	28.36	−0.48	NAM	Nu
*PeNAC*036	ZX.01G0072730	1	74366801:74368257−	972	323	5.95	37.35	−0.68	NAM	Nu
*PeNAC*037	ZX.01G0088890	1	145808892:145810179−	948	315	5.66	35.35	−0.54	NAM	Nu
*PeNAC*038	ZX.01G0098050	1	200585364:200587337+	798	265	5.39	30.65	−0.84	NAM	Nu
*PeNAC*039	ZX.01G0100880	1	207999819:208002937−	1659	552	6.27	61.65	−0.57	NAM	Nu
*PeNAC*040	ZX.01G0103760	1	211866169:211868051−	951	316	6.15	35.90	−0.68	NAM	Nu
*PeNAC*041	ZX.01G0105360	1	214023833:214025399+	489	162	9.05	19.13	−1.00	NAM	Nu
*PeNAC*042	ZX.01G0106110	1	214570314:214571882+	1089	362	8.64	40.83	−0.57	NAM	Nu
*PeNAC*043	ZX.01G0108420	1	216133135:216134149+	987	328	9.08	37.88	−0.61	NAM	Nu
*PeNAC*044	ZX.01G0119560	1	224899411:224899995+	366	121	9.88	13.82	−0.58	NAM	Nu
*PeNAC*045	ZX.01G0119950	1	225148150:225149359−	948	315	7.73	35.94	−0.67	NAM	Nu
*PeNAC*046	ZX.01G0124150	1	230489426:230490776+	1107	369	7.69	42.61	−0.69	NAM	Nu
*PeNAC*047	ZX.01G0133730	1	262377111:262378939−	909	302	7.58	34.89	−0.79	NAM	Nu
*PeNAC*048	ZX.01G0133750	1	262394524:262395244−	636	211	8.19	24.85	−1.09	NAM	Nu
*PeNAC*049	ZX.02G0000540	2	1467235:1468705+	753	250	8.6	28.86	−0.52	NAM	Nu
*PeNAC*050	ZX.02G0002530	2	13108643:13111176+	1086	361	7.96	40.60	−0.62	NAM	Nu
*PeNAC*051	ZX.02G0007450	2	40672265:40674447−	1080	359	7.67	41.21	−0.88	NAM	Nu
*PeNAC*052	ZX.02G0014000	2	58641330:58659908−	4677	1558	5.07	171.85	−0.35	NAM	Nu
*PeNAC*053	ZX.02G0014390	2	59415565:59420569−	1281	426	8.02	48.59	−0.45	NAM	Nu
*PeNAC*054	ZX.02G0019640	2	63478416:63482864−	828	275	5.8	31.21	−0.76	NAM	Nu
*PeNAC*055	ZX.02G0019780	2	63666086:63671972+	1392	463	6.43	50.89	−0.39	NAM	Nu
*PeNAC*056	ZX.02G0025580	2	84602549:84605064−	1047	348	9.03	39.44	−0.70	NAM	Nu
*PeNAC*057	ZX.02G0025650	2	84960535:84963308−	786	261	8.99	29.95	−0.66	NAM	Nu
*PeNAC*058	ZX.02G0033670	2	137938703:137941490−	960	319	7.68	36.47	−0.60	NAM	Nu
*PeNAC*059	ZX.03G0009360	3	59729802:59733176−	1824	607	5.17	66.96	−0.63	NAM	Nu
*PeNAC*060	ZX.03G0014870	3	87367329:87372056+	2058	685	9.42	78.44	−0.46	NAM	Nu
*PeNAC*061	ZX.04G0001790	4	3669240:3674336−	1404	467	6.57	52.87	−0.99	NAM	Nu
*PeNAC*062	ZX.04G0007290	4	15276359:15278415−	1044	347	7.16	38.60	−0.45	NAM	Nu
*PeNAC*063	ZX.04G0008230	4	19174946:19177149−	1065	354	8.85	39.14	−0.62	NAM	Nu
*PeNAC*064	ZX.04G0008250	4	19259346:19259614+	1083	360	9.61	40.04	−0.34	NAM	Nu
*PeNAC*065	ZX.04G0031700	4	114534441:114535578+	363	120	9.11	14.07	−0.76	NAM	Nu
*PeNAC*066	ZX.05G0001190	5	5955648:5958062−	1014	337	5.48	38.81	−0.72	NAM	Nu
*PeNAC*067	ZX.05G0007500	5	55621532:55623758−	819	272	9.02	31.29	−0.72	NAM	Nu
*PeNAC*068	ZX.05G0020700	5	113741000:113749751+	3600	1199	4.96	134.58	−0.64	NAM	Nu
*PeNAC*069	ZX.05G0020740	5	113793918:113819533+	6591	2196	5.46	247.30	−0.56	NAM	Nu
*PeNAC*070	ZX.05G0020790	5	113905506:113906809+	555	184	9.35	21.49	−0.70	NAM	Nu
*PeNAC*071	ZX.06G0004520	6	23459916:23460583+	522	173	9.79	20.06	−0.50	NAM	Nu
*PeNAC*072	ZX.06G0004540	6	23511513:23513832−	927	308	8.14	34.83	−0.82	NAM	Nu
*PeNAC*073	ZX.06G0005330	6	29554361:29555622+	1038	345	8.7	38.03	−0.64	NAM	Nu
*PeNAC*074	ZX.06G0015410	6	89341553:89343399+	927	308	7.27	34.58	−0.75	NAM	Nu
*PeNAC*075	ZX.06G0021470	6	106964012:106965215−	771	256	9.18	29.60	−0.66	NAM	Nu
*PeNAC*076	ZX.07G0003310	7	25510023:25511478−	1203	400	8.03	44.56	−0.47	NAM	Nu
*PeNAC*077	ZX.07G0008780	7	63523916:63529200−	1722	573	4.59	63.69	−0.47	NAM	Nu
*PeNAC*078	ZX.07G0008800	7	63533831:63537472−	1341	446	5.82	49.81	−0.66	NAM	Nu
*PeNAC*079	ZX.07G0014550	7	89809350:89810935+	822	273	8.18	31.64	−0.77	NAM	Nu
*PeNAC*080	ZX.07G0021160	7	106496308:106497670+	1020	340	6.52	39.48	−0.75	NAM	Nu
*PeNAC*081	ZX.07G0022510	7	111057642:111060055+	1308	435	8.55	48.88	−0.45	NAM	Nu
*PeNAC*082	ZX.08G0005930	8	29016171:29020373−	1284	427	5.23	48.14	−0.87	NAM	Nu
*PeNAC*083	ZX.08G0010990	8	37491750:37495892−	822	273	5.53	31.21	−0.55	NAM	Nu
*PeNAC*084	ZX.08G0013270	8	41401775:41404960+	801	266	6.25	30.06	−0.48	NAM	Nu
*PeNAC*085	ZX.08G0015100	8	44473207:44475253+	957	318	8.44	35.76	−0.57	NAM	Nu
*PeNAC*086	ZX.08G0021410	8	51373292:51382406−	4956	1651	6.18	187.80	−0.15	NAM	Ch*
*PeNAC*087	ZX.08G0024000	8	53222928:53224185+	705	234	9.81	26.54	−0.66	NAM	Nu
*PeNAC*088	ZX.08G0025970	8	54197073:54199731−	1470	489	9.33	58.40	−0.74	NAM	Nu
*PeNAC*089	ZX.08G0026690	8	54675834:54688752+	5028	1675	5.35	186.83	−0.49	NAM	Nu
*PeNAC*090	ZX.08G0027600	8	55299803:55300975−	576	191	5.11	22.01	−0.47	NAM	Nu
*PeNAC*091	ZX.08G0027810	8	55381204:55383785−	885	294	8.51	33.50	−0.75	NAM	Nu
*PeNAC*092	ZX.08G0042450	8	104087526:104088787−	945	314	8.98	35.80	−0.58	NAM	Nu
*PeNAC*093	ZX.08G0042690	8	104851354:104853500−	885	294	6.97	33.15	−0.63	NAM	Nu
*PeNAC*094	ZX.09G0002850	9	18300989:18303584−	1029	342	8.62	37.83	−0.58	NAM	Nu
*PeNAC*095	ZX.09G0005830	9	31170610:31174757+	1476	491	6.53	55.35	−0.81	NAM	Nu
*PeNAC*096	ZX.09G0008400	9	34008540:34010727−	987	338	9.01	37.57	−0.59	NAM	Nu
*PeNAC*097	ZX.09G0014050	9	40874598:40876610+	831	276	8.44	31.67	−0.32	NAM	Nu
*PeNAC*098	ZX.09G0014090	9	40922963:40930277+	1587	528	5.69	59.99	−0.44	NAM	Nu
*PeNAC*099	ZX.09G0014220	9	41025163:41027169−	1014	337	5.81	38.31	−0.52	NAM	Nu
*PeNAC*100	ZX.09G0014490	9	41524588:41527202+	765	254	9.16	28.68	−0.68	NAM	Nu
*PeNAC*101	ZX.09G0017690	9	51685705:51688515−	1194	397	9	43.76	−0.60	NAM	Nu
*PeNAC*102	ZX.09G0018660	9	55884796:55886910+	1086	361	7.21	40.52	−0.51	NAM	Nu
*PeNAC*103	ZX.09G0019090	9	60302611:60304137+	1029	419	6.25	47.33	−0.66	NAM	Nu
*PeNAC*104	ZX.09G0019120	9	60428293:60429979+	1218	405	5.97	45.77	−0.77	NAM	Nu
*PeNAC*105	ZX.09G0021380	9	74779640:74783392+	1791	596	5.23	65.60	−0.63	NAM	Nu

Cr*, chromosome NO; O.R.F*, open reading frame; a.a*, amino acid/protein length; M.W*, molecular weight (KDa); pI*, isoelectric point; GRAVY*, grand average of hydropathicity; SCL*, sub-cellular localization*-Nucleus; Ch*, chloroplast. Positive (+) and negative (−) signs represent the presence of a gene on the positive and negative strands of that marker at the genome location.

### Phylogenetic analysis of *PeNAC* genes

**FIGURE 1:**
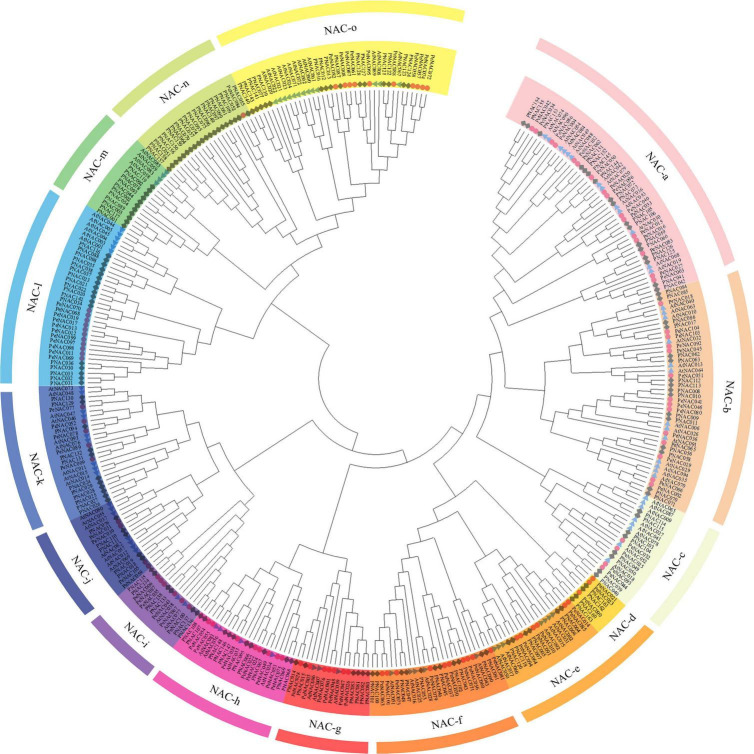
An unrooted neighbor-joining (NJ) phylogenetic tree based on NAC protein sequence alignment between *A. thaliana* (AtNAC), *P. edulis* (PeNAC), and *P. trichocarpa* (PNAC) with 1000 bootstraps. All the NAC members were divided into 15 groups and presented in different colors. The blue triangle represents AtNAC sequences, the red circle represents PeNAC sequences and the gray square represents PNAC sequences.

To access the evolutionary relationship between the *NAC* genes, a neighbor-joining (NJ) phylogenetic tree was generated by MEGA software using the NAC protein sequences of passion fruit (PeNAC), *Arabidopsis* (AtNAC), and *P. trichocarpa* (PNAC) (the protein sequences of all NACs used in the phylogenetic tree have been provided in Supplementary Table 2). The phylogenetic tree was divided into fifteen groups and named as NAC-a to NAC-o according to the previous publication on *P. trichocarpa PNACs* (Hu et al., 2010; Figure 1). The results showed that *PeNAC* genes were as diverse as NAC proteins, and *PeNAC* genes were found to be unevenly distributed across all groups. All groups contained PeNAC members except the NAC-m group, which only consisted of AtNAC and PNAC members (Figure 1). The NAC-a group had a maximum of 16 PeNAC members, followed by NAC-b with 14 PeNAC members, and NAC-n was the smallest group with only one PeNAC member. Notably, the *PeNAC* members aligned with *AtNAC* and *PNAC* genes in one group, showing their similarity to *Populus* and *Arabidopsis NAC* genes and may exhibit the same function, From the phylogenetic tree analysis, the NAC-m group contained only *AtNAC* and *PNAC* members but no *PeNAC* gene, whereas, NAC-n group contained only *PeNAC* and *PNAC* members but no *AtNAC* gene, indicating that some genes have undergone evolutionary changes, and these *NAC* genes may develop in passion fruit or lost in *P. trichocarpa* and *Arabidopsis* during evolution (Figure 1). The *NAC* genes play a key role in a variety of biological and functional studies, so phylogenetic analysis provides a better understanding of *NAC* genes. Taken together, genes in one group with co-adaptive associations and relationships might have the same functions and require further function studies.

### Gene structure and motif analysis of *PeNAC* genes

**FIGURE 2:**
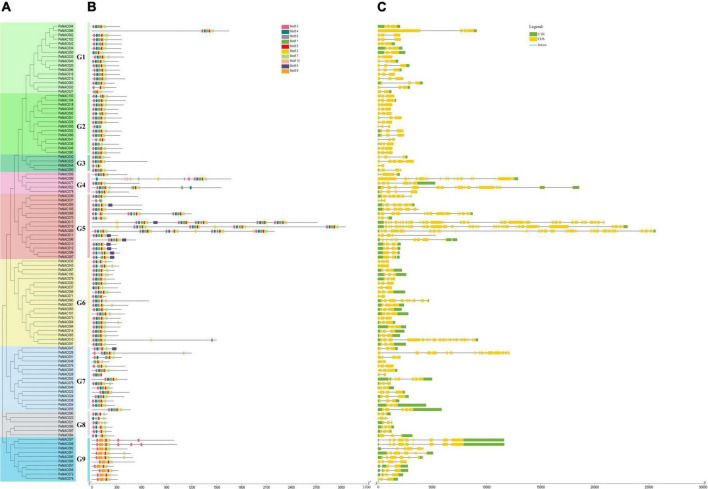
The unrooted phylogenetic tree, conserved motifs, and gene structure of *PeNAC* genes. **(A)** The neighbor-joining tree on the left comprised 105 PeNAC proteins, different colors represented different groups. **(B)** Conserved motifs were represented *via* boxes and different colors represent different motifs. **(C)** *PeNAC* genes structures, yellow color indicates the exons, the green color shows the untranslated 5′ and 3′-regions, and gray color indicates the introns.

To investigate the relationship between all 105 *PeNAC* genes, a phylogenetic tree was constructed by the NJ method and divided into 9 subclades (G1 to G9) (Figure 2). The results showed that G6 was the major group with 20 PeNAC members, and G3 was the smallest group with only 4 PeNAC members (Figure 2A). The *PeNAC* genes conserved motifs and gene structures (exon-intron) were further investigated. Moreover, 10 conserved motifs were recognized among all 105 PeNAC proteins by MEME online tool (Supplementary Table 3 and Figure 2B). Motif prediction results revealed that the highly conserved PeNAC members may have similar motif information and that the *N*-terminal motif contains A-E subdomains that confer DNA-binding activity (Figure 2B).

The motif results revealed that there were at least 3 to 8 conserved motifs in all the 105 PeNAC proteins, of which the most prominent motifs were Motifs 1, 2, 3, 4, 5, 6, and 7. Among all 105 PeNAC proteins, the proteins including PeNAC011, PeNAC012, PeNAC013, PeNAC059 PeNAC097, PeNAC098, and PeNAC099 contained a maximum of 8 motifs, followed by PeNAC003, PeNAC04, and PeNAC044 proteins contained 4 motifs, whereas the PeNAC031 protein contained at least 3 motifs (Figure 2B). Among the groups, the G1, G2, G3, G4, G6, and G7 groups were most consistent with nearly similar motifs (Motifs 1–7), while the G5, G8, and G9 groups were different from other groups containing Motif 8 (G5), Motif 9 (G9), and Motif 10 (G8 and G9) respectively (Figures 2A,B). The conserved motif results are consistent with the phylogenetic relationship, as members with similar conserved motifs are grouped in the same phylogenetic clade and might have similar functions (Figures 2A,B).

**FIGURE 3:**
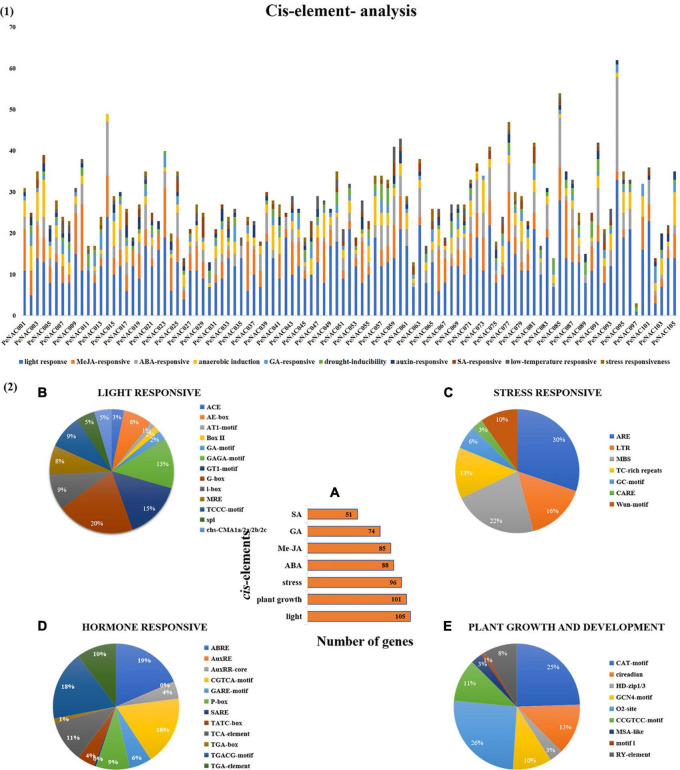
The *cis*-regulatory element analysis of *PeNAC* genes **(A)**. (A) The sum number of *PeNAC* genes involved in four categories of *cis*-elements and the percentage (%) ratio of the numerous *cis*-elements from each category is presented in pie charts; **(B)** light-responsive; **(C)** stress-responsive; **(D)** hormone-responsive and **(E)** plant growth and development. Different colors indicate different *cis*-elements and their ratios present in *PeNAC* genes.

Furthermore, the gene structure results showed that the number of introns found in the *PeNAC* gene varied from 1 and 38 (Figure 2C). The phylogenetic results (Figure 2A) showed that the genes within the same clade had a similar number of intron-exon. For example, in the G1–G2 (30 genes) and G6 (20 genes) groups, most PeNAC members have 3 exons (Figure 3C). In groups G7-G8, there were 2–8 exons, while in the G5 group, the number of exons varied from 3 to 31. Briefly, the coding regions were diverse, ranging from 2 to 39 in the *PeNAC*, with an average of 2 introns per gene (Figure 2C). The maximum number of 38, 35, and 31introns were found in *PeNAC019*, *PeNAC017*, and *PeNAC069* genes. The *PeNAC027*, *PeNAC043*, *PeNAC044*, *PeNAC048*, and *PeNAC071* genes have only one intron (Figure 2C). The results from gene structure analysis provide consistent validation to confirm our phylogenetic classification among *PeNAC* genes (Figure 2). Taken together, the results of phylogenetic relationship, group classification, conserved motif, and gene structure analyses suggested that the proteins of *PeNAC* genes were highly conserved and genes within the same group might perform similar functions, but it requires further investigations.

### *Cis* element analysis of *PeNAC* genes

The *cis*-regulatory element analysis of the *PeNAC* genes was performed to further understand the possible roles of *PeNAC* genes in response to plant growth and development, phytohormone, and light and stress responsiveness (Figure 3). Furthermore, the *cis*-regulatory element main categories were divided into 10 sub-categories of *cis*-elements and presented in Figure 3.1. A total of 2,862 *cis*-elements belonging to different categories were identified in 105 *PeNAC* genes. The highest *cis*-elements 45% (1,297/2,862) were found in light-responsive category followed by MeJA-responsive 15% (436/2862), ABA-responsive 11% (318/2862), anaerobic induction 10% (275/2,862), drought-responsive 4% (107/2,862), GA-responsive 4% (123/2862), low-temperature responsive 3% (72/2,862), SA-responsive 3% (80/2862), auxin-responsive 3% (97/2862), and minimum *cis*-elements were found in stress-responsive (drought, wound and pathogen, biotic and abiotic stresses, etc.) 2% (57/2,862) category (Figure 3.1).

The light-responsive *cis*-elements include ACE, AE-box, AT1-motif, Box II, GA-motif, GAGA-motif, GTI-motif, G-box, I-box, MRE, TCCC-motif, spI, and cbs-CMA1a/2a/2b/2c (Figure 3.2B). G-box covered the maximum (20%) and ATI-motif (1%) minimum light-responsive *cis*-elements (Figure 3.2B). The stress-related *cis*-element include ARE, LTR, MBS, TC-rich repeats, GC-motif, CARE, and wun-motif. ARE *cis*-elements covering a maximum of 30% and CARE minimum of 3% stress-responsive category, respectively (Figure 3.2C). Hormone responsive categories include ABRE (abs*cis*ic acid-responsive), AuxRE and AuxRR-core (auxin response element), CGTCA-motif, GARE-motif, P-box, TCA-element, and SARE (salicylic acid-responsive elements), TATC-box (gibberellin responsive) (salicylic acid-responsive), TGA-box, TGA element and TGACG-motif (MeJA responsive). The largest hormone-responsive elements included ABRE (19%), TGACG-motif (18%), and the smallest 1% of TGA-box (Figure 3.2D). Plant growth and development-related *cis*-element include CAT-motif, circadian, HD-zip1/3, GCN4-motif, O2-sit, CCGTCC-motif, MSA-like, motifI and RY-element. This category has a maximum of 26% O2-sit and a minimum 1% motif-1 *cis*-elements (Figure 3.2E).

In addition, *PeNAC* genes were classified according to the number of genes involved in each category and it was found that 105 *PeNAC* genes were involved in light-responsive, 101 genes in metabolic (plant growth and development), and 96 genes in stress-responsive (drought, wound, biotic/abiotic stresses, etc.) *cis*-elements. *PeNAC* genes were also associated with different hormone response classes, including 88 genes in ABA, 85 genes in MeJA, 74 genes in GA, and 51 genes in SA response *cis*-elements (Figure 3.2A). In conclusion, the responses of different *PeNAC* genes to different *cis*-regulatory elements indicated that the transcription profiles of *PeNAC* genes differed in different *cis*-elements and further functional studies are needed (The details information on *cis*-elements in *PeNAC* genes has been provided in Supplementary Table 4).

### Chromosomal location and collinearity analysis of *PeNAC* genes

**FIGURE 4:**
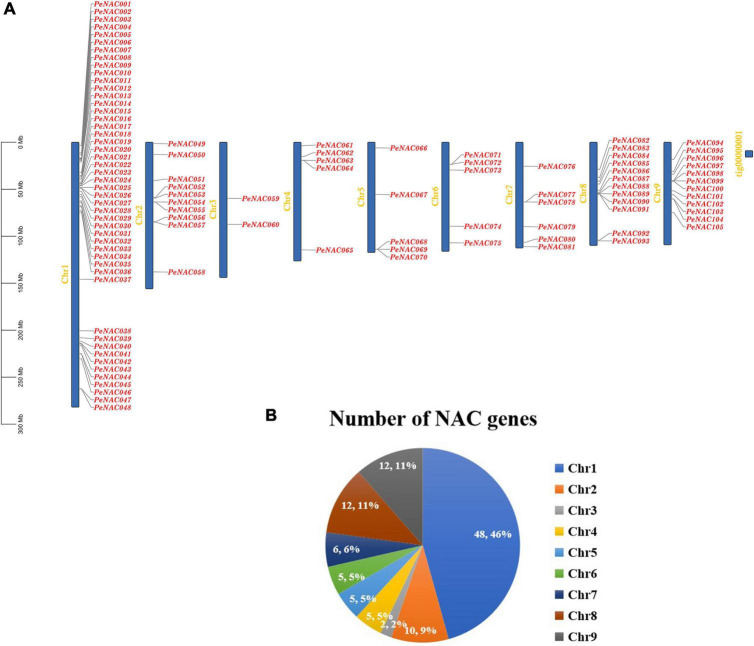
Genomic location of *PeNAC* genes on passion fruit chromosomes. **(A)** Chromosomal location of *PeNAC* genes, the scale represents the 300MB chromosomal distance, and the *PeNAC* genes are represented in red color. **(B)** The sum number of *PeNAC* genes appeared on nine passion fruit chromosomes and the percentage (%) ratio of *PeNAC* genes from each chromosome is presented in pie charts; different colors indicate a different number of *PeNAC* genes and their ratio present in the chromosome of passion fruit.

**FIGURE 5:**
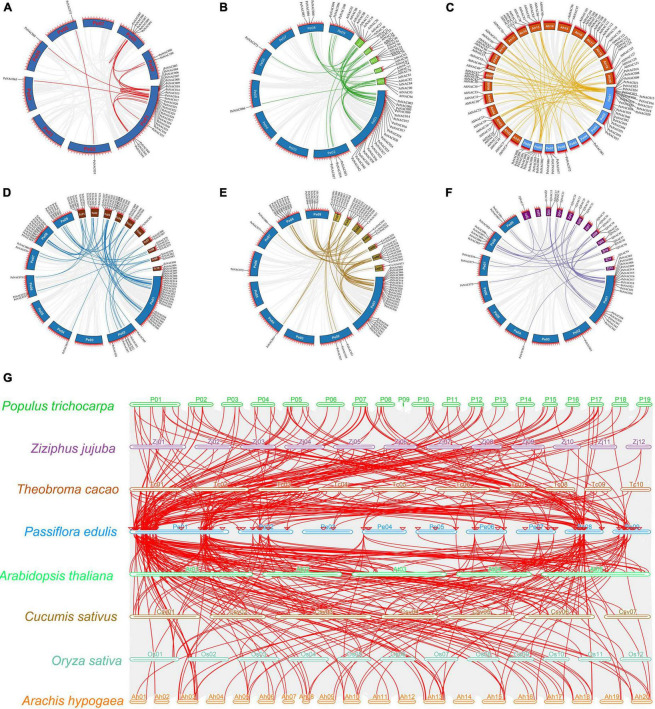
Circos illustrations of the *PeNAC* genes duplication and multicollinearity analysis of *NAC* genes between *P. edulis*, *A. thaliana*, *P. trichocarpa*, *Z. jujuba*, *T. cacao*, *C. sativus*, *O. sativa*, and *A. hypogaea*. **(A)** Gene duplication of *NAC* genes in *P. edulis*, the background gray lines show all syntenic blocks in the passion fruit genome, and the red lines show the segmental or tandem duplication line regions among-*PeNAC* genes. The approximate location of *PeNAC* genes is labeled with a short gray line outside the chromosome with gene names. **(B–F)** Orthologous of *P. edulis NAC* genes with *A. thaliana* (*AtNAC*), *A. hypogaea* (*AhNAC*), *T. cacao* (*TcNAC*), *C. sativa* (*CsvNAC*), and *Z. jujuba* (*ZjNAC*). Chromosomes of *P. edulis* are presented with Pe01–Pe09, *A. thaliana* with At01–At05, *A. hypogaea* with Ah01–Ah20, *T. cacao* with Tc01–TC10 and *Z. jujuba* with Zj01–Zj12. The background gray lines show all the syntenic blocks genomes of different species, five colors lines represented the syntenic between *P. edulis* and other species *NAC* genes. **(G)** Multicollinearity analysis of *NAC* genes between *P. edulis*, *A. thaliana*, *P. trichocarpa*, *Z. jujuba*, *T. cacao*, *C. sativus*, *O. sativa*, and *A. hypogaea* species, the gray lines in the background represent the collinear blocks within *P. edulis* and other species genomes.

The chromosomal locations of *PeNAC* genes were studied based on passion fruit genome DNA sequence annotations. The results discovered that the 105 *PeNAC* genes were unequally distributed on all nine passion fruit chromosomes (Figure 4A). The maximum number of *PeNAC* genes were appeared to be on chromosome number one (48 genes) followed by chromosomes numbers eight and nine (12 genes); chromosome number two (10 genes); chromosome number seven (6 genes); and chromosomes four to six (every 5 genes). Chromosome number two contained the least only two *PeNAC* genes (Figure 4B). Gene duplications including tandem and/or segmental greatly contribute to the diversity and evolutionary history of gene families and play an important role in understanding the adaptive evolution of species. The gene duplication results revealed that, out of 105 *PeNAC* genes, there were 23 *PeNAC* orthologous gene pairs (Figure 5A). Among the 23 *PeNAC* orthologous gene pairs, 11 gene pairs were located on chromosomes number one as tandem duplicated, while 12 gene pairs were located on different chromosomes as segmental duplicated. Only one duplication gene pair was found on chromosomes two, four, five, and six, whereas no gene pair was found on chromosomes three and seven (Figure 5A). These results suggest that segmental and tandem duplication happened during the *PeNAC* genes evolution.

Additionally, the Ka/Ks ratios were calculated to access the selection pressure and divergence rates between *PeNAC* duplicated genes (Supplementary Table 5). Generally, the Ka/Ks > 1 indicates that the gene underwent positive selection, Ka/Ks < 1 indicates negative purification selection and Ka/Ks = 1 as neutral selection. The results of Ka/Ks showed that all the duplicated *PeNAC* genes have a Ka/Ks < 1 (0.12 to 0.47) representing that all duplicated genes underwent purifying selection (Supplementary Table 5). Moreover, the divergence rate among duplicated *PeNAC* genes was measured and it was estimated to be between 6.62 to 221.92 million years ago (Supplementary Table 5). The results showed that the divergence rate between duplicated *PeNAC* genes was estimated to be between 6.62 and 221.92 million years ago (Supplementary Table 5).

Furthermore, a comprehensive collinear analysis was performed among the *NAC* genes of the following species including *P. edulis* (*PeNAC*), *A. thaliana* (*AtNAC*), and *Z. jujuba* (*ZjNAC*), *T. cacao* (*TcNAC*), *A. hypogaea* (*AhNAC*), and *C. sativa* (*CsvNAC*). Overall, a total of 284 *NAC* gene pairs were identified in all the above species, of which, the highest number of *NAC* gene pairs was found between *PeNAC-AhNAC* (86 gene pairs) followed by *PeNAC-CsvNAC* (63 gene pairs), *PeNAC-TcNAC* (61 gene pairs), *PeNAC-AtNAC* (42 gene pairs), and the lowest (32 gene pairs) among *PeNAC-ZjNAC* (Figures 5B–F and Supplementary Table 6). Moreover, the results of the collinear analysis revealed that 31 *PeNAC* genes were paired with 32 *AtNAC* genes (Figure 5B), 38 *PeNAC* genes were paired with 61 *AhNAC* genes (Figure 5C), 50 *PeNAC* genes were paired with 38 *TcNAC* genes (Figure 5D), 43 *PeNAC* genes were paired with 37 *CsvNAC* genes (Figure 5E), and 31 *PeNAC* genes were paired with 23 *ZjNAC* genes (Figure 5F). Among the *NAC* gene collinearity in all the above species, the *NAC* genes of *P. edulis* were highly collinear with *A. hypogaea NAC* than other species, indicating that they may belong to same ancestor with similar functions, but this requires further studies.

In addition, a multicollinearity analysis was achieved to reveal the robust orthologs of *P. edulis NAC* genes in the genomes of seven other species including *A. thaliana*, *Z. jujuba*, *T. cacao*, *A. hypogaea, P. trichocarpa*, *O. sativa*, and *C. sativa* (Figure 5G). The collinear gene pairs of the above species were found to be inferred and have undergone lineage-specific expansion during evolution. The results showed that the highest collinearity was found between *P. edulis* and *P. trichocarpa* (132 orthologs) followed by *P. edulis* and *A. hypogaea* (100 orthologs), *P. edulis* and *T. cacao* (78 orthologs), *P. edulis* and *C. sativa* (68 orthologs), *P. edulis* and *A. thaliana* (66 orthologs), *P. edulis* and *Z. jujuba* (41 orthologs), and least collinearity was found between *P. edulis* and *O. sativa* (19 orthologs), respectively (Figure 5G and Supplementary Table 7). By comparing the *P. edulis* chromosomes, the *P. edulis* chromosome one shared the largest orthologs with all other species. Overall, the maximum collinear orthologs were found between *P. edulis* and *P. trichocarpa* indicating that the *NAC* genes were conserved and may share the same ancestors except for duplication or loss of the *NAC* genes.

### Protein–protein interaction of PeNAC

**FIGURE 6:**
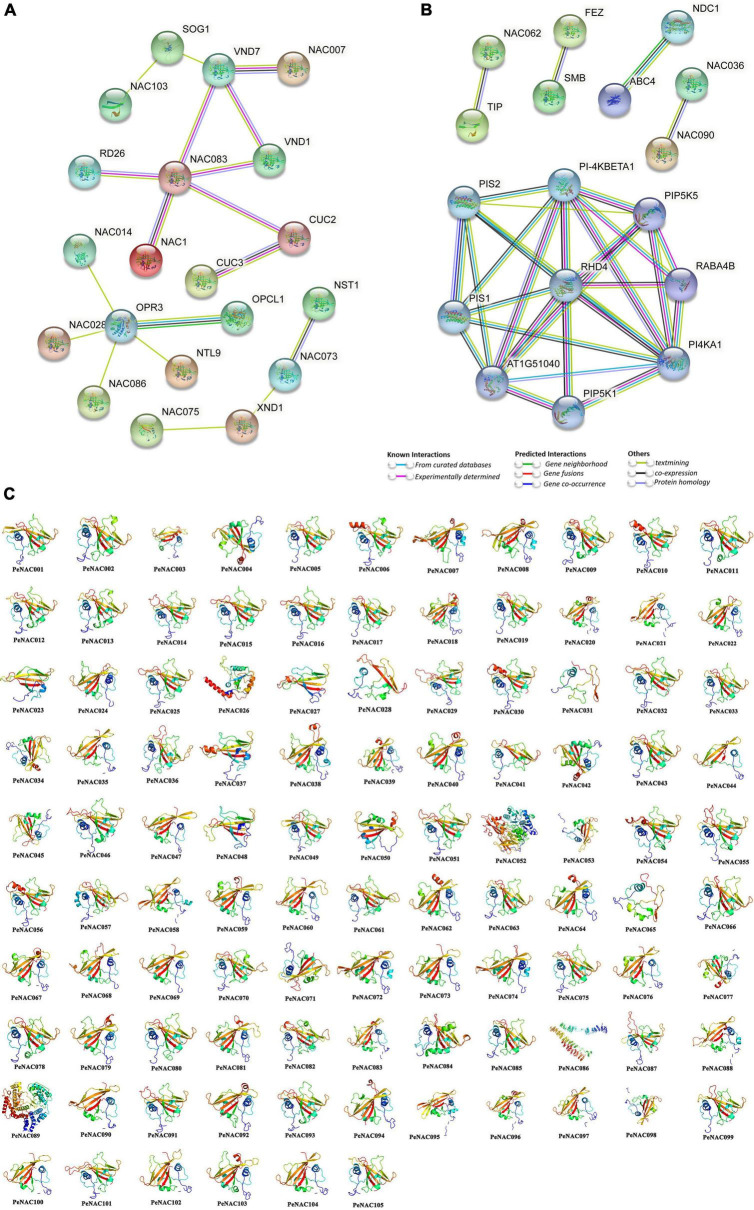
Protein–protein interaction and predicted 3D models of PeNAC proteins. **(A)** Medium confidence interaction (0.4). **(B)** High confidence interaction (0.7). **(C)** 3D models of PeNAC proteins were constructed using the online Phyre2 server with default mode.

The PeNAC protein interaction network based on *Arabidopsis* protein orthologs was performed, and PeNAC proteins that were highly similar to *Arabidopsis* proteins were called STRING proteins. All 105 PeNAC proteins interacted with known *Arabidopsis* proteins and PeNAC proteins present in different groups may have different functions (Figures 6A,B and Supplementary Table 8). PeNAC036, PeNAC041, PeNAC046, and PeNAC080 were homologous with AtNAC007 and interacts with AtVND1/7 (VASCULAR-RELATED NAC-DOMAIN1/7) protein. PeNAC035, PeNAC043, PeNAC067, and PeNAC100 were homologous with AtNAC083 and have a strong interaction with AtRD26/AtNAC72, ATVND1/7, AtNAC1, and AtCUS2/3 proteins. PeNAC057, PeNAC058, PeNAC072, PeNAC074, and PeNAC088 were homologous with AtNAC073 and interact with AtNST1 (NAC SECONDARY WALL THICKENING PROMOTING FACTOR) and AtXND1 (xylem NAC domain 1) proteins (Figure 6A). PeNAC068, PeNAC069, and PeNAC105 were homologous with AtNAC014, PeNAC025 was homologous with AtNAC028, PeNAC013 was homologous with AtNAC086 and they interact with a common protein that is AtOPR3 (OXOPHYTODIENOATE-REDUCTASE 3) (Figure 6A).

PeNAC070 was homologous with AtNAC062 and interact with AtTIP (TCV-INTERACTING PROTEIN) (Figure 6B). PeNAC038, PeNAC054, and PeNAC055 were homologous with AtNAC090 and PeNAC049, PeNAC053, and PeNAC075 were homologous with AtNAC036 and they have a strong interaction between them (Figure 6B). PeNAC089 was homologous with AtPI-4KBETA1 and have a strong interaction with AtPIS1/2, AtPIP5K1/5, AtPI4KA1, and AT1G51040 (Figure 6B and Supplementary Table 8). Furthermore, protein–protein interaction results are inconsistent with phylogenetic relationships because PeNAC proteins align with AtNAC proteins found in the same phylogenetic group, for example, PeNAC062 and PeNAC102 were homologous with AtNAC83 and interact with AtCUC2/3 proteins and present in the same phylogenetic group (G1). Additionally, the 3D structures of all the 105 PeNAC proteins were predicted using an online Phyre2 server with the reference model templates including c4d0mW, c1xi4D, c3ni2A, c3ulxA, c4rciC, and d1ut7a. Overall, up to 49% (52/105) and 47% (49/105) of PeNAC proteins were modeled with the c3ulxA and d1ut7a reference templates. However, only single proteins including PeNAC052, PeNAC086, and PeNAC089 were predicted to be modeled with the c3ni2A, c1xi4D, and c4d0mW reference templates (Figure 6). All 105 PeNAC proteins showed similar 3D structures and they have flexible structures due to the presence of coils (Figure 6). The PeNAC 3D results suggest that NAC proteins may be ancestrally similar to each other from individual genomes, or preliminary adjustments and might be stabilized during long-term domestication leading to changes in protein structures and functions.

### Prediction of putative micro-RNAs directing *PeNAC* genes

**FIGURE 7:**
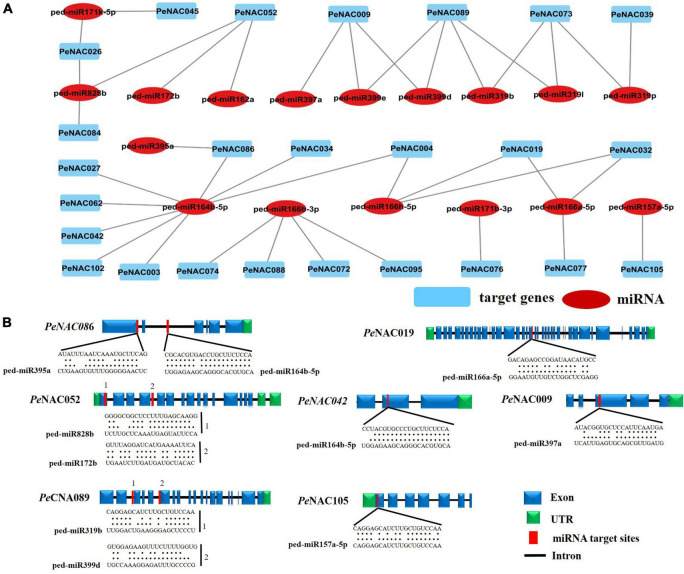
Predicted miRNAs targeting *PeNAC* genes. **(A)** Network illustration of predicted miRNA targeted *PeNAC* genes. Blue rectangle shapes represent the predicted miRNAs and red oval shapes represent targeted *PeNAC* genes. **(B)** The schematic diagram indicates the *PeNAC* genes targeted by miRNAs and the putative miRNAs sites are indicated by red color, upper sequences are from the gene region and lower sequences are from the miRNAs.

To better understand the regulatory mechanisms of miRNAs in *PeNAC* genes, a total of 17 miRNAs (ped-miRNAs) were identified, that belong to 11 different miRNA families. The network interactions and schematic diagram of miRNA targeting sites in *PeNAC* genes are presented in Figures 7A,B. As shown in Figure 7A, the 17 identified miRNA targeted 25 *PeNAC* genes and results showed that ped-miR164b-5p targeted the most 8 *PeNAC* genes (*PeNCA003*, *PeNCA004*, *PeNAC027*, *PeNCA034*, *PeNCA042*, *PeNCA062*, and *PeNCA086*) followed by ped-miR166b-3p targeted 4 *PeNAC* genes (*PeNCA072*, *PeNCA074*, *PeNCA088*, and *PeNCA095*), whereas ped-miR171b-3p, ped-miR319p, and ped-miR157a-5p targeted at least one *PeNAC076*, *PeNAC039*, and *PeNAC105* gene respectively. Compared between the targeted genes, *PeNAC089* was targeted the most by 4 miRNAs including ped-miR319l, ped-miR319b, ped-miR3199d, and ped-miR3199e. *PeNAC009* was targeted by 3 miRNAs including ped-miR399d, ped-miR399e, and ped-miR397a; PeNAC052 was targeted by 3 miRNAs including ped-miR162a, ped-miR172b, and ped-miR828B; similarly, *PeNAC073* was also targeted by 3 miRNAs including ped-miR319p, ped-miR319I, and ped-miR319b (Figure 7A and Supplementary Table 9). In conclusion, the ped-miR164b-5p and ped-miR166b-3p were the major miRNAs, while *PeNAC089*, *PeNAC009*, *PeNAC052*, and *PeNAC073* were the major targeted genes that may play an important role and require further functional studies (Figure 7 and the detail information about *PeNAC* target genes and miRNAs have been provided in Supplementary Table 9).

### Transcription factor regulatory network analysis of *PeNAC* genes

**FIGURE 8:**
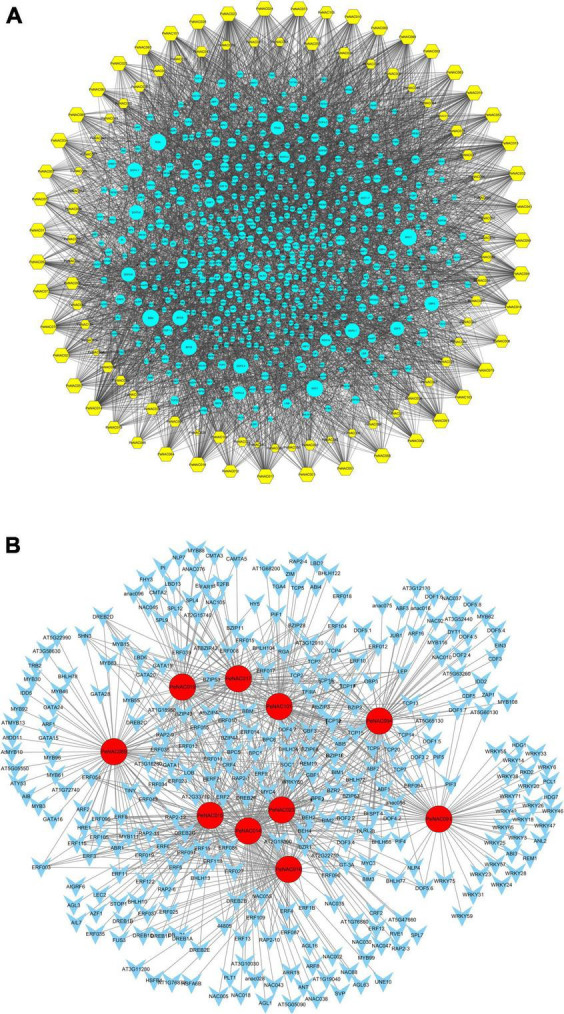
The putative transcription factor regulatory network analysis of *PeNAC* genes. **(A)** Turquoise circular nodes represent transcription factors; yellow hexagonal nodes represent *PeNAC* genes and node size represents the degree of interaction between nodes based on degree value. **(B)** The top 10 highly enriched and targeted *PeNAC* genes are shown, darker the color shows highly enriched. Transcriptional factors were predicted by PTRM online program and a regulatory network was constructed using the Cytoscape 3.9 software.

The potential TFs were investigated in the upstream regions of all 105 *PeNAC* genes and a TF regulatory network was constructed using Cytoscape. The results showed that among all 105 *PeNAC* genes, a total of 7,029 TFs were identified, belonging to 43 different TF families including BBR-BPC, AP2, bHLH, bZIP, Dof, NAC, WRKY, TCP, ERF, GRAS, MYB, C2H2, and B3 (Figure 8 and Supplementary Table 10). The predicted TF families revealed that ERF (1344 members) was highly enriched followed by BBR-BPC (864 members), Dof (814 members), NAC (530 members), MIKC- MADS (458 members), and bZIP (404 members) (Figure 8 and Supplementary Table 10). The least enriched families were also predicted to contain only a few members including VOZ (1 member), LFY (2 members), GRF (3 members), SRS (4 members), WOX (7 members), and EIL (9 members). Among all 105 *PeNAC* genes, PeNAC089 was the most targeted by 299 TFs followed by *PeNAC094* (240 TFs), *PeNAC023* (221 TFs), *PeNAC014* (219 TFs), and *PeNAC074* (167 TFs). Whereas, *PeNAC068* was targeted the least by only 4 TFs (Figure 8 and Supplementary Table 10).

The *PeNAC* genes were targeted by various types and numbers of TF families, for example, *PeNAC089* was enriched in ERF (186), MYB (32), GATA (25), and BBR-BPR (14) family members, *PeNAC094* was enriched in Dof (64), bZIP (33), and bHLH (32) family members, *PeNAC023* was enriched in ERF (82), BBR-BPC (39), and TCP (28) family members. The TF interaction networks of all 105 *PeNAC* genes are shown in Figure A. The 10 most enriched *PeNAC* genes with TFs are shown in Figure B (Supplementary Table 10). TFs related to plant growth, development, and response to biotic and abiotic stress were also found in *PeNAC* genes including ERF, TCP, bHLH, BBR-BPC, WRKY, bZIP, MYB, and AP2 respectively (Figure 8 and Supplementary Table 10).

### Gene ontology and Kyoto encyclopedia of genes and genomes annotation analysis of *PeNAC* genes

**FIGURE 9:**
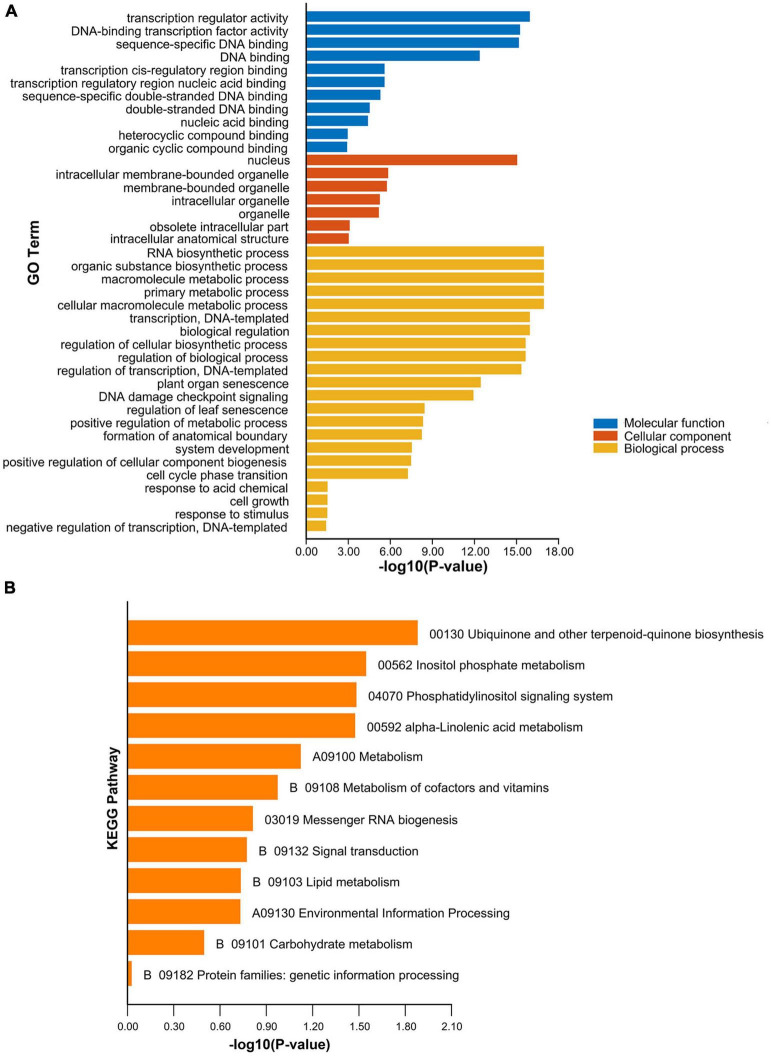
Gene ontology and KEGG enrichment analysis of *PeNAC* genes. **(A)** The highly enriched GO terms in *PeNAC* genes. **(B)** The highly enriched KEGG pathways in *PeNAC* genes. Details about GO annotation enrichment and terms of MF, CC, BP, and KEGG pathways can be found in Supplementary Tables 11, 12.

The GO and KEGG annotation and enrichment analysis were conducted to further understand *PeNAC* genes at the molecular level and classified into the biological process (BP), cellular component (CC), and molecular function (MF) (Figure 9 and Supplementary Tables 11, 12). Overall, the highest 628 terms were found in GO-BP class followed by 55 terms in GO-CC and the lowest 38 terms were found in the GO-MF 38-MF class. The high enrichment analysis revealed that there were 11 highly enriched terms in GO-MF class including transcription regulator activity (GO:0140110, 76 genes), DNA-binding transcription factor activity (GO:0003700, 76 genes), sequence-specific DNA binding (GO:0043565, 33 genes), DNA binding (GO:0003677, 33 genes), nucleic acid binding (GO:0003676, 33 genes), heterocyclic compound binding (GO:1901363, 33 genes), organic cyclic compound binding (GO:0097159, 33 genes), transcription *cis*-regulatory region binding (GO:0000976, 14 genes), transcription regulatory region nucleic acid binding (GO:0001067, 14 genes), sequence-specific double-stranded DNA binding (GO:1990837, 14 genes), and double-stranded DNA binding (GO:0003690, 14 genes) (Figure 9A).

In the GO-CC classification, 7 of the 55 terms were highly enriched including intracellular membrane-bounded organelle (GO:0043231, 39 genes), membrane-bounded organelle (GO:0043227, 39 genes), intracellular organelle (GO:0043229, 39 genes), organelle (GO:0043226, 39 genes), obsolete intracellular part (GO:0044424, 39 genes), intracellular anatomical structure (GO:0005622, 39 genes), and nucleus (GO:0005634, 37 genes). Whereas in GO-BP class, 15 terms were highly enriched including organic substance biosynthetic process (GO:1901576, 78 genes), primary metabolic process (GO:0044238, 78 genes), biological regulation (GO:0065007, 78 genes), regulation of biological process (GO:0050789, 77 genes), RNA biosynthetic process (GO:0032774, 76 genes), macromolecule metabolic process (GO:0043170, 76 genes), cellular macromolecule metabolic process (GO:0044260, 76 genes), transcription, DNA-templated (GO:0006351, 76 genes), regulation of cellular biosynthetic process (GO:0031326, 76 genes), regulation of transcription, DNA-templated (GO:0006355, 76 genes), response to stimulus (GO:0050896, 46 genes), positive regulation of biological process (GO:0048518, 35 genes), system development (GO:0048731, 34 genes), plant organ senescence (GO:0090693, 14 genes), and response to light intensity (GO:0009642, 10 genes) (Figure 9A and Supplementary Table 11). In conclusion, the GO results indicate that the BP category was highly enriched followed by the CC and finally the MF category.

Furthermore, KEGG pathway enrichment analysis of 105 *PeNAC* genes showed that 12 pathways were predicted to be involved in different functions (Figure 9B and Supplementary Table 12). As shown in Figure 9B, among the predicted KEGG pathways, the highly enriched pathways include ubiquinone and another terpenoid-quinone biosynthesis (00130), inositol phosphate metabolism (00562), phosphatidylinositol signaling system (04070), alpha-Linolenic acid metabolism (00592), metabolism (A09100), and metabolism of cofactors and vitamins (B09108). In conclusion, the GO and KEGG analysis suggest that *PeNAC* gene may play important functions in different biological, molecular, and cellular processes including different biotic and abiotic stresses, metabolism, and biosynthetic responses related to hormones but require further studies. The details of GO and KEGG annotation results and numerically significantly enriched terms for MF, CC, and BP have been provided in Supplementary Tables 11A,B, 12.

### Expression analysis of *PeNAC* genes in different fruit developmental stages

**FIGURE 10:**
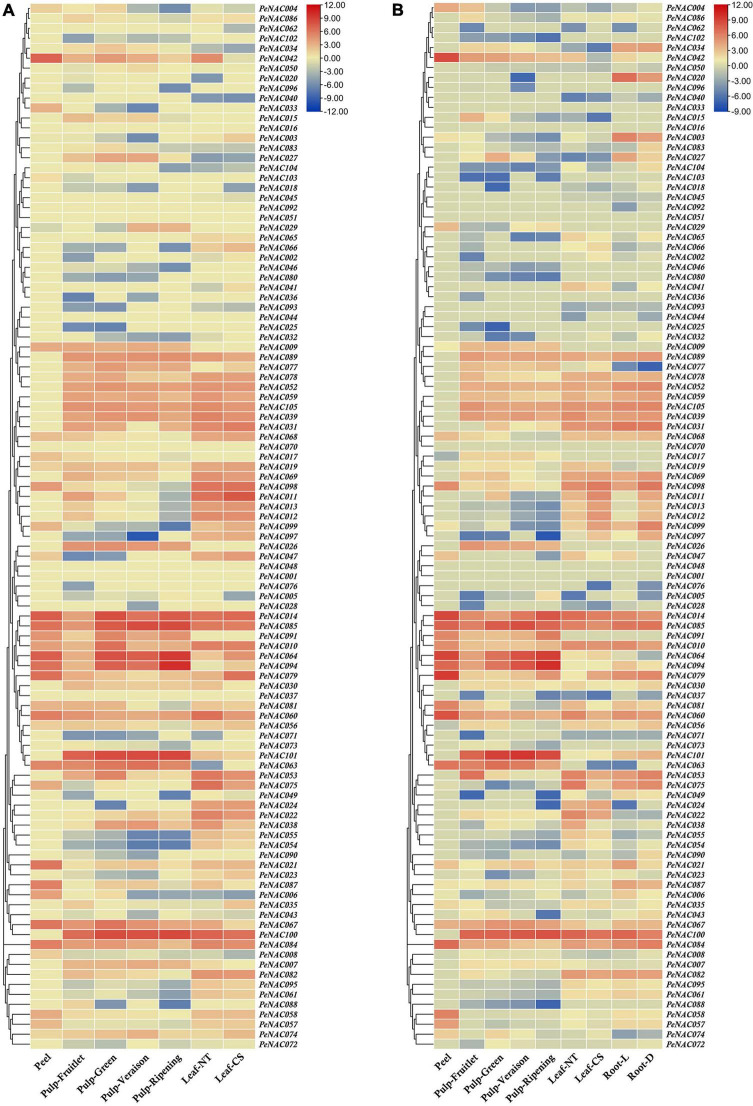
The heatmap showing the expression profiles of *PeNAC* genes in pulp, root, peel, and leave tissues of yellow and purple passion fruit cultivars under different conditions. **(A)** *PeNAC* expressions in the peel, pulp (Fruitlet, Green, Veraison, and Ripening stages) and leaf (NT and CS) of yellow passion fruit. **(B)** *PeNAC* genes expressions in the peel, pulp (Fruitlet, Green, Veraison, and Ripening stages), leaf (NT and CS) and root (L and D). The NT and CS indicate the normal temperature (NT) and chilling stress (CS) conditions. L and D represent the samples from limestone (L) and sandy dolomite (D) rocky desertification areas. Fragments per kilobase per million (FPKM) values of *PeNAC* genes in all tissues were transformed by log2 and a heatmap was constructed by TBtools software (the red color shows the highest and the blue color shows the lowest expression levels in the expression bar).

The expression profiles of all 105 *PeNAC* genes in the pulp tissue of yellow and purple passion fruit at fruitlet, green, veraison, and ripening stages were evaluated based on FPKM values. FPKM values were converted to log^2^FC and were displayed into heatmap by TBtools software (FPKM and log^2^FC values have been provided in Supplementary Table 13). The *PeNAC* genes showed a diverse expression in both cultivars at different fruit developmental stages. The result showed that, overall, 90% (95/105) and 87% (92/105) *PeNAC* genes were either positively or negatively expressed during fruit development in both cultivars. The following genes were not expressed in both cultivars including *PeNAC001*, *PeNAC013*, *PeNAC016*, *PeNAC017*, *PeNAC035*, *PeNAC040*, *PeNAC041*, *PeNAC044*, *PeNAC050*, and *PeNAC098* suggesting that they may not play a role during fruit development. Moreover, *PeNAC014*, *PeNAC063*, *PeNAC064*, *PeNAC085*, *PeNAC094*, *PeNAC100*, and *PeNAC101* genes had the highest expression levels (FC ≥ 5) during fruit developmental stages of both cultivars (Figure 10 and Supplementary Table 13).

**FIGURE 11:**
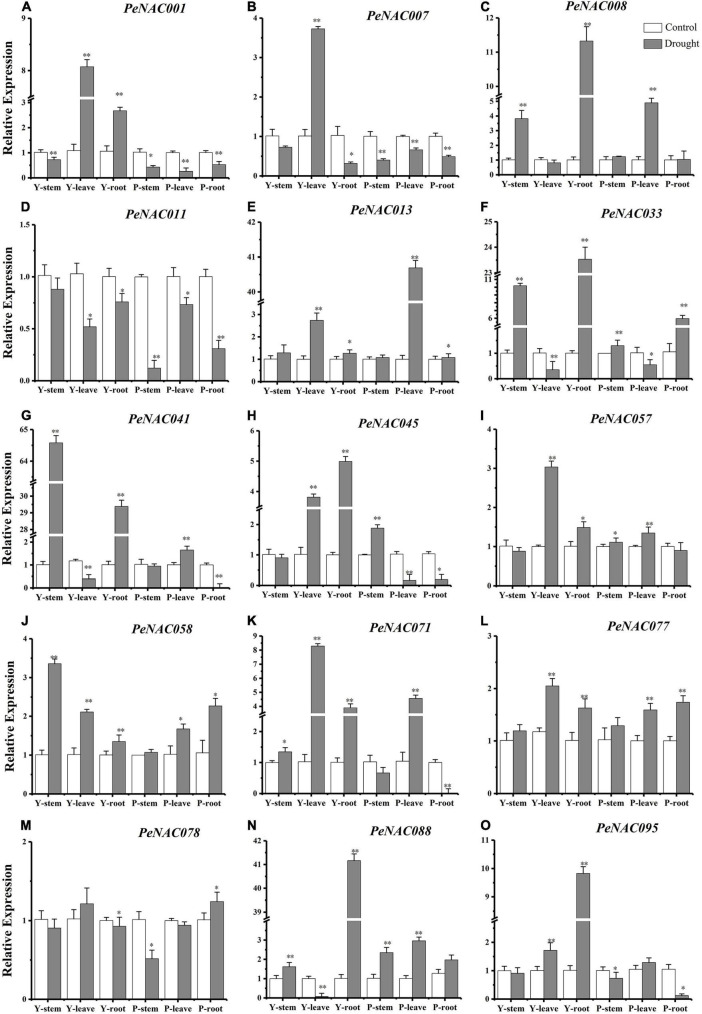
The relative expressions of *PeNAC* genes in the stem, root, and leave tissues of yellow and purple passion fruits under drought stress and control conditions **(A–O)**. The relative gene expression levels were calculated using the 2^–ΔΔCt^. Vertical bars represent means ± SD (*n* = 3). The * and ** show significance at *p* ≤ 0.05 and *p* ≤ 0.01. Y represents the yellow passion fruit; P represents the purple passion fruit; and dpi represents the days post-inoculation.

In the yellow fruit, there were 80% *PeNAC* (84/105), 82% *PeNAC* (87/105), 73% *PeNAC* (77/105), and 68% *PeNAC* (72/105) genes were expressed in fruitlet, green, veraison and ripening stages. In comparison between different stages, the ripening stage had the highest expression 9.99 FC (*PeNAC094*) followed by veraison stage 8.41 FC (*PeNAC100*), green stage 7.28 FC *(PeNAC100*), and fruitlet stage 8.36 FC (*PeNAC104*) respectively. There were 35 *PeNAC* genes (40%) with ≥2 FC expression levels at the green stage, followed by 33 *PeNAC* genes (39%) at the fruitlet stage, 29 *PeNAC* genes (37%), and the lowest of 25 *PeNAC* genes (34%) at ripening stage of yellow fruit (Figure 11A and Supplementary Table 13).

In purple fruit, the expression results showed that 75% *PeNAC* (79/105), 83% *PeNAC* (88/105), 73% *PeNAC* (77/105), and 75% *PeNAC* (79/105) genes were expressed in fruitlet, green, veraison and ripening stages. In the comparison of different stages, the ripening stage had the highest expression 10.07 FC *(PeNAC94*) followed by veraison stage 9.61 FC (*PeNAC100*), fruitlet stage 9.16 FC (*PeNAC100*), and green stage 7.44 FC *(PeNAC100*) respectively. A maximum of 35 *PeNAC* genes (44%) had ≥2 FC expression levels in the fruitlet stage, followed by 33 *PeNAC* genes (37%) in the green stage, 24 *PeNAC* genes (31%) in the veraison stage, and a minimum of 22 *PeNAC* (27%) at the ripening stage of purple fruit (Figure 10B and Supplementary Table 13). In conclusion, overall, the purple *PeNAC* genes had the highest expressions compared with yellow fruit. The ripening stage had the highest expression compared with other developmental stages. The genes including *PeNAC014*, *PeNAC063*, *PeNAC064*, *PeNAC085*, *PeNAC094*, *PeNAC100*, and *PeNAC101* with constant and highest expressions (≥5 FC) in all developmental stages of both cultivars suggested that these genes may play important roles in fruit development, which can be used for further functional studies.

### Expression analysis of *PeNAC* genes in different tissues

The *PeNAC* genes expressions in roots of purple, leave and peel tissues of yellow and purple passion fruits were detected based on FPKM expression values. The FPKM expression values were converted to log^2^FC and visualized into a heatmap (FPKM and log^2^FC values have been provided in Supplementary Table 14). All 105 *PeNAC* genes showed as diverse expressions in different tissues of both cultivars. The expression result showed that among 105 *PeNAC* genes, overall, 85% (90/105) *PeNAC* genes were expressed in all tested tissues, of which, 80% (84/105) genes were expressed in yellow and purple leaves, 79% (83/105) *PeNAC* were expressed in yellow and purple root, and only 33% (35/105) *PeNAC* genes were expressed in the yellow and purple peel. Peel tissue had the highest expression levels *(PeNAC079*, 9.42 FC) followed by leaves (*PeNAC011*, 7.84 FC) and root (*PeNAC100*, 7.38 FC), whereas, the genes including *PeNAC001*, *PeNAC016*, *PeNAC025*, *PeNAC026*, *PeNAC036*, *PeNAC044*, *PeNAC046*, *PeNAC048*, *PeNAC051*, *PeNAC070*, *PeNAC073*, *PeNAC080*, *PeNAC088*, *PeNAC096*, and *PeNAC102* were not expressed in all tissues suggesting that they may not play role under these conditions (Figures 10A,B and Supplementary Table 14).

In yellow and purple leave tissues under CS and NT conditions, the expression results showed that 77% *PeNAC* (81/105), 74% *PeNAC* (78/105), 72% (76/105), and 71% (75/105) genes were expressed in purple leaf-NT, purple leaf-CS, yellow-leaf-NT, and yellow leaf-CS conditions. In comparison between cultivars, the yellow leaf-NT had the highest expression 7.84 FC (*PeNAC011*) followed by purple leaf-NT 7.41 FC (*PeNAC100*), yellow leaf-NT 7.20 FC (*PeNAC100*), and purple leaf-CS 6.80 FC (*PeNAC001*). A maximum of 42 *PeNAC* genes (55%) had ≥2 expression levels in yellow leaf-NT followed by 41 *PeNAC* genes (50%) in purple leaf-NT, 37 *PeNAC* genes (49%) in yellow leaf-CS, and a minimum of 34 *PeNAC* genes (43%) in the yellow leaf-NT conditions (Figure 10A and Supplementary Table 14). In purple root tissues under L and D conditions, the results showed that 78% *PeNAC* (82/105) and 77% *PeNAC* (81/105) genes were expressed in purple roots under L and D conditions. Purple root under the L condition has the highest expression 7.38 FC (*PeNAC100*) compared with D condition 6.72 FC (*PeNAC085*). Purple root under D condition had a maximum of 40 *PeNAC* genes (49%) with ≥2 FC expression compared with L condition 37 *PeNAC* genes (45%) (Figure 10B and Supplementary Table 14).

In yellow and purple peel tissue, the expression results showed that 33% *PeNAC* (35/105) and 31% *PeNAC* (33/105) genes were expressed in yellow and purple peel respectively. Purple peel had the highest expression 9.42 FC (*PeNAC079*) compared with yellow peel 7.55 FC (*PeNAC014*), whereas among the expressed genes, yellow peel had the highest number of PeNAC genes 25 (71%) with ≥2 FC expression levels than purple peel *PeNAC* genes 23 (69%) (Figures 10A,B and Supplementary Table 14). Taken together, the results showed that the highest number of genes were expressed in leave tissues followed by root and least in peel tissue, while purple peel tissue had the highest expression levels (*PeNAC079*, 9.42 FC) followed by yellow leaves-CS (*PeNAC011*, 7.84 FC) and purple root-L (*PeNAC100*, 7.38 FC) conditions. The genes including *PeNAC011*, *PeNAC014*, *PeNAC042*, *PeNAC079*, *PeNAC085*, *PeNAC094*, and *PeNAC100*, etc., with ≥6 FC expression levels in all tissues of both cultivars indicate that these genes might have important roles in regulating mechanism and can be used for further functional studies.

### Expression analysis of *PeNAC* genes under drought stress

It has been reported that *NAC* genes play a crucial role under drought stress conditions and the expression levels of *NAC* genes under drought stress conditions. The qRT-PCR expression profiles of selected *PeNAC* genes in stems, roots, and leave tissues of yellow and purple passion fruit under drought stress conditions were investigated (Figure 11). The results revealed that, overall, in both, all the tested *PeNAC* genes exhibited increased expression profiles under drought stress conditions compared with controls except the *PeNAC0011* gene (Figure 11). Among tissues and cultivars, the significantly highest expressions were found in Y-stem (*PeNAC041* > 64-fold, Figure 11G) followed by Y-root (*PeNAC088* > 40-fold, Figure 11N), P-leave (*PeNAC013* > 40-fold, Figure 11E), and Y-root (*PeNAC008* and *PeNAC033* > 11-fold, Figures 11C,F), whereas the downregulated/lowest expressions in all tissues of both cultivars were found in *PeNAC011* and *PeNAC078* genes (Figures 11D,M) respectively. In *PeNAC095* (Figure 11O), the expression in yellow root tissue was significantly higher (>9-fold) than in yellow and purple roots, leaves, and stems. In comparison to the tissues of both cultivars, the expressions of *PeNAC001* (Figure 11A) and *PeNAC007* (Figure 1B) genes were significantly down-regulated in all purple tissues under drought conditions compared to yellow and controls.

**FIGURE 12:**
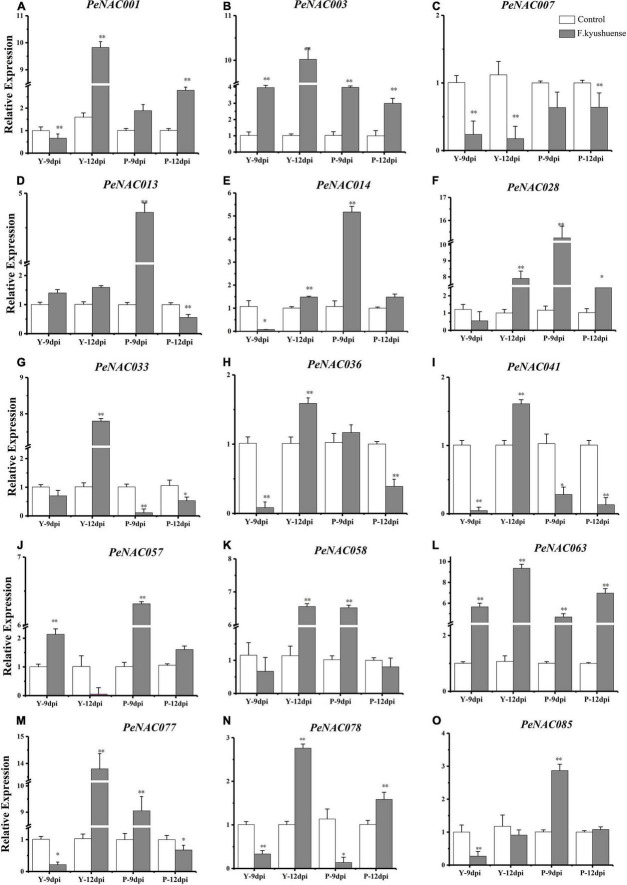
The relative expressions of *PeNAC* genes in peel tissues of yellow and purple passion fruits under *F. kyushuense* fungal biotic stress and control conditions **(A–O**). The relative gene expression levels were calculated using the 2^–ΔΔCt^. Vertical bars represent means ± SD (*n* = 3). The* and ** show significance at *p* ≤ 0.05 and *p* ≤ 0.0. Y represents the yellow passion fruit; P represents the purple passion fruit; and dpi represents the days post-inoculation.

The expression levels of the *PeNAC077* gene in all tissues were relatively stable, ranging from 1 to 2-fold (Figure 12L). Compared with controls, the expressions of most of the tested *PeNAC* genes such as *PeNAC013* (Figure 12E), *PeNAC033* (Figure 12F), *PeNAC41* (Figure 12G), *PeNAC88* (Figure 12N), and *PeNAC095* (Figure 12O) showed an obvious upward trend under drought stress conditions. In conclusion, the expression levels of yellow passion fruit tissues were generally higher than purple tissue under drought stress conditions compared with controls (Figure 12). Genes such as *PeNAC008*, *PeNAC013*, *PeNAC033*, *PeNAC041*, and *PeNAC088* were highly expressed (>10-fold) in passion fruit (Figure 12), suggesting that these genes may play an important role in passion fruit drought stress and provide evidence for further functional studies.

### Expression analysis of *PeNAC* gene under *F. kyushuense* stress conditions

The possible role of *PeNAC* genes in yellow and purple passion fruit under biotic stress by a pathogenic fungus (*F. kyushuense*) at qRT-PCR expression levels was investigated (Figure 11). The results revealed that, overall, in both cultivars, all the tested *PeNAC* genes exhibited increased expression profiles under fugal biotic stress conditions compared with controls, except for the *PeNAC007* gene (Figure 11). The tested *PeNAC* genes showed different expression levels in both cultivars at 9 dpi and 12 dpi compared to controls. In comparison of both cultivars; in yellow passion fruit, the expression profiles of *PeNAC* genes at 12 dpi were significantly higher than that at 9 dpi including *PeNAC001* (Figure 11A), *PeNAC003* (Figure 11B), *PeNAC033* (Figure 11G), *PeNAC036* (Figure 11H), *PeNAC041* (Figure 11I), *PeNAC063* (Figure 11L), *PeNAC077* (Figure 11M), and *PeNAC078* (Figure 11N). However, in purple fruits, the expression levels of *PeNAC* genes were generally higher at 9 dpi than at 12 dpi including *PeNAC013* (Figure 11D), *PeNAC014* (Figure 11E), *PeNAC028* (Figure 11F), *PeNAC057* (Figure 11J), and *PeNAC085* (Figure 11O), respectively.

Taken together, these results suggest that *PeNAC* genes in purple fruits were more active at 9 dpi fungal infection, whereas in yellow fruits were more active after a longer infection time at 12 dpi. There were significant differences in the expression levels of almost all *PeNAC* genes compared with controls, with a clear upward trend under *F. kyushuense* infection except *PeNAC007* (Figure 11C). The *PeNAC007* might not play a role under biotic stress conditions in passion fruit. Genes such as *PeNAC001*, *PeNAC003, PeNAC028, PeNAC033, PeNAC057, PeNAC058, PeNAC063*, and *PeNAC077* were highly upregulated (log2FC > 5, Figure 11) under *F. kyushuense* stress conditions in passion fruit, indicating that these genes may play an important role in the resistance of passion fruit to biotic stresses and provide evidence for further exploration of functional studies.

## Discussion

Plant TFs are DNA-binding proteins that play an important role in different plant processes (Baillo et al., 2019). To date, different TF families have been identified and functionally characterized on genome levels in different plant species. NAC is one of the largest TF families that involves in the regulation of plant senescence, metabolism, development, morphogenesis, signal transduction, and stress responses (Singh et al., 2021). Genome-wide *NAC* gene family has been reported in numerous plant species and members of *NAC* have been functionally characterized in different plants including *Arabidopsis* (Tran et al., 2004), rice (Ooka et al., 2003), barley (Murozuka et al., 2018), maize (Wang G. et al., 2020), and peanut (Li P. et al., 2021) respectively. However, there is no comprehensive information regarding the *NAC* gene family in passion fruit. In this study, a total of 105 *NAC* genes were identified and characterized in the passion fruit genome (Table 1). All 105 *NAC* genes were unequally distributed on all nine-passion fruit chromones, while chromosome one contained the maximum number of *NAC* genes (46%) (Table 1 and Figure 4).

It has been reported that within the same gene family, the distribution of genes on different chromosomes may be due to their involvement in multiple functions (Ahmad et al., 2021). The 105 *PeNAC* genes identified in the passion fruit genome were almost similar to the number of *NAC* genes in apricot (102 *NAC*) (Xu et al., 2021), pepper (104 *NAC*) (Diao et al., 2018), and cacao (102 *NAC*) (Shen et al., 2019), while some species have a larger number of *NAC* genes including rice (151 *NAC*) (Ooka et al., 2003), poplar (163 *NAC*) (Hu et al., 2010), apple (180 *NAC*) (Su et al., 2013), and barley (167 *NAC*) (Murozuka et al., 2018), whereas some species have a smaller number of *NAC* genes including maize (87 *NAC*) (Wang G. et al., 2020), pitaya (64 *NAC*) (Hu et al., 2022), and eggplant (90 *NAC*) (Wan et al., 2021).

The diversity in the number and structural features of the same gene family members in different species may be due to differences in genome size and long-term plant evolution (Wang P. et al., 2018). All 105 *PeNAC* genes contained NAM domains and are consistent with previous reports on *Arabidopsis* apple and rice (Tran et al., 2004; Gao et al., 2010; Saad et al., 2013; Su et al., 2013), moreover, Trupkin et al. (2019) mentioned that genes containing NAM domains involved in plant growth and development. All the 105 *PeNAC* genes were predicted to localize to the nucleus (Table 1) and Duan et al. (2017) reported that TFs localized to the nucleus activate the responses including the reactive oxygen species homeostasis, osmolyte production and detoxification under different stresses. A phylogenetic tree was constructed using *A. thalian* (AtNAC), *P. trichocarpa* (PNAC), and *P. edulis* (PeNAC) proteins and divided into 15 groups (NAC-a to NAC-o) based on *P. trichocarpa* PNACs (Hu et al., 2010). In the G1 and G5 groups, 16 and 15 PeNAC proteins were orthologous to AtNAC019 and AtNAC056, respectively, suggesting that PeNAC proteins may play a role in drought tolerance, fruit ripening, hormone signaling, and secondary wall synthesis in the plant (Rohrmann et al., 2012; Wang J. et al., 2018). In the G2 group, 14 PeNAC proteins were homologous to AtNAC091, and these NAC proteins may be involved in membrane and plant stress associated (Sun et al., 2013). In the NAC-I, NAC-j, and NAC-k groups, 15 and 11 PeNAC proteins were orthologous with AtNAC003, AtNAC008, and AtNAC069, respectively, and these NAC proteins were involved in the DNA damage response, cold, heat, drought tolerance stresses, and development (Liu et al., 2013; Miao et al., 2021).

The conserved motif results revealed that there were at least 3 to 8 conserved motifs (Motif 1–8) in all of 105 PeNAC proteins (Figure 2B), indicating that PeNAC proteins have a remarkably conserved protein structure. These results are consistent with the findings of apricot and pitaya NAC by Xu et al. (2021), Hu et al. (2022), which also reported a distinct number of conserved motifs. Moreover, the majority of the conserved motifs were present in the N-terminus of PeNAC and Xu et al. (2021) mentioned that the N-terminus is an important part of the NAC proteins, as previously shown in potato (Koch et al., 2000). Most of the *PeNAC* had three exons or two introns (Figure 2C) and genes within the same phylogenetic clade (Figure 2A) have a similar number of exons. These results are consistent with pitaya (Hu et al., 2022), kiwifruit (Jia et al., 2021), and cucumber (Liu et al., 2018) *NAC* genes suggesting that the genetic makeup of passion fruit *NAC* genes are similar with the previously reported species.

The *PeNAC cis*-regulatory element analysis identified distinct plant developmental and stress response elements (Figure 3). The following *cis*-elements including ACE, G-box, AE-box, ARE, LTR, MBS, ABRE, TGACG-motif, and CAT-motif were found abundantly in *PeNAC* genes indicating that *PeNAC* may play an important role in plant development, hormones, and stress responses. Logemann and Hahlbrock (2002) demonstrated the important role of ACE in pathogen defense. Wang C. et al. (2020) reported that ACE promoted the response to ethylene stress and inhibition of ethylene. Liu et al. (2016) mentioned that the G-box participates in light-responsive processes. Maruyama et al. (2012) reported that ABRE *cis*-acting elements regulate the expression of dehydration- and salt-responsive genes in *Arabidopsis* and rice. Kaur et al. (2017) reported that genes containing MBS *cis*-element play an important role under drought stress. Levasseur and Pontarotti (2011) mentioned that gene duplication is the basis for the generation of new genes and functions, and is a major driving force in the evolution of genomes and gene families.

The *PeNAC* gene duplication analysis was performed and found less than 1 Ka/Ks values, indicating a purifying selection with 6.62 to 221.92 mya duplication process between tandem and segmentally duplicated *PeNAC* gens. These results are in constant with preceding reports such as Jin et al. (2017) reported that the *NAC* gene family experienced a divergence rate of 145 mya in dicotyledonous and grass plants, furthermore, Tuskan et al. (2006) reported that a whole-genome duplication occurred in *Populus* at 60–65 mya. Moreover, 12 *PeNAC* gene pairs were segmental duplicated and 11 gene pairs were tandem duplicated, which are in consistent with Li X. et al. (2021) and Li P. et al. (2021) reports, who also found the segmental and tandem duplicated genes in genome-wide *NAC* family analysis. In addition, a comprehensive syntenic analysis of *P. edulis NAC* with *A.* thaliana, Z. *jujuba*, *T. cacao*, *A. hypogaea*, and *C. sativa NAC* identified 284 *NAC* gene pairs, of which, the maximum 86 *NAC* gene pairs were between *P. edulis* and *A. hypogaea* (38 *PeNAC*-61 *AhNAC*).

Moreover, multicollinearity analysis of *P. edulis NAC* genes found the highest collinearity with *P. trichocarpa* (132 orthologs) followed by *A. hypogaea* (100 orthologs). These results are consistent with Ma et al. (2021) that the *P. edulis* genome maximum likelihood analysis revealed a close relationship between *P. edulis* and *P. trichocarpa* orthologous genes, indicating that these orthologous may share the same ancestors and retain corresponding functions. The protein–protein interaction network analysis provides information for specific gene family functions associated with known proteins (Piya et al., 2014). In present study protein–protein interaction analysis revealed that most of the PeNAC proteins have homology and strong interactions with known *Arabidopsis* proteins including AtNAC083, AtNAC086, AtRD26, AtVND1/7, AtNAC1, AtNAC007, AtCUS2/3, AtNAC073, AtNST1, AtXND1, AtNAC014, and AtNAC028 suggesting that PeNAC may perform similar functions. The highest homology was found between PeNAC035, PeNAC043, PeNAC067, PeNAC100, and AtNAC083 proteins which are consistent with previous reports. For example, it has been reported that AtNAC083 is a crucial transcriptional activator that play a role in response to salt stress response, leaf aging process, and xylem vessel formation (Suyal et al., 2014).

Wen-Fang et al. (2015) proposed that NAC83 might play an important role in response to abiotic stresses in pomelo (*Citrus maxima*). Yao et al. (2020) reported that *NAC86* is involved in stress responses in *Populus simonii × P. nigra*. Le Hénanff et al. (2013) results provide evidence that VvNAC1 regulates grapevine development and stress responses to pathogens in the grapevine (*Vitis vinifera*). Protein 3D structure prediction is recognized as a reliable analytical technique to better understand the protein molecular functions (Kelley et al., 2015). In the current study, most of the PeNAC proteins showed similar 3D structures with the reference templates such as c3ulxA, which is considered as a DNA binding protein and annotated as stress-induced NAC1 in rice (Chen et al., 2011) and d1ut7a template annotated as a NAC domain (Ernst et al., 2004). The current findings are consistent with Wang et al. (2021), who also reported similar 3D structures of NAC in *Chrysanthemum nankingense*. The 3D modeling results discovered that PeNAC proteins possessed similar 3D structures, indicating that PeNAC proteins may belong to similar ancestors or underwent purifying selection to keep stabilization during the long-term evolution after the initial divergence (Zhu et al., 2019).

It is well known that under specific circumstances, miRNAs can directly or indirectly activate and repress gene expression by playing an important role in the regulation of gene expression (O’Brien et al., 2018). In recent years, the identification and functional characterization of miRNAs have been reported in numerous plant species including *Arabidopsis* (Palatnik et al., 2007; Allen et al., 2010), rice (Jiang et al., 2019), *Brassica napus* (Wen et al., 2021), passion fruit (Paul et al., 2020), soybean (*Glycine max*) (Song et al., 2011), and apple (Kaja et al., 2015), which were involved in different metabolism, development, and environmental stresses. In current study, 17 ped-miRNAs belonging to 11 different miRNAs families were identified that targeted 25 *PeNAC* genes. The ped-miR164 family targeted the most eight *PeNAC* genes and has been reported to be involved in abiotic stress in *P. euphratica* (Lu et al., 2017), drought stress in rice (Fang et al., 2014), the resistance of wheat to stripe rust (Feng et al., 2014), and fruit ripening (Wang W. Q. et al., 2020). The ped-miR166 family targeted four *PeNAC* genes and has been reported to play important role in response to abiotic and biotic stresses in soyabean (Li et al., 2017), shoot apical meristem and floral development in *Arabidopsis* (Jung and Park, 2007), cold stress in tomato (Valiollahi et al., 2014) and cadmium tolerance and accumulation in rice (Ding et al., 2018).

Moreover, the ped-miR171b-3p, ped-miR319p, and ped-miR157a-5p targeted at least one *PeNAC* gene. Huang et al. (2019) reported that miR171 and miR319 play a crucial role in citrus adaptation to long-term B toxicity by targeting MYB. He et al. (2018) reported that miR157 plays important role in shoot morphogenesis of *Arabidopsis*, iron deficiency responses in tomato (Zhu et al., 2022), and fruit development in pineapple (*Ananas comosus*) (Yew and Kumar, 2011). These findings suggested that the identified ped-miRNAs may play vital roles in combating multiple functions in plant development and stresses by altering the transcriptional level of *PeNAC* genes, however further functional studies are needed. Plant TFs play an important role in plant growth, development, and response to different stresses (Joshi et al., 2016; Sharif et al., 2021). Different TFs were identified in the promoter regions of *PeNAC* genes including BBR-BPC, AP2, bHLH, bZIP, Dof, WRKY, ERF, and MYB (Figure 8). The highly enriched TF families were ERF, BBR-BPC, MIKC-MADS, and bZIP respectively. Xie et al. (2019) mentioned that AP2/ERF is involved in hormone and stress responses in *Arabidopsis*. Wen et al. (2016) stated that Dof plays significant role in plant growth, development, and responses to stresses. Theune et al. (2019) reported that BBR-BPC is involved in flower development, size of the stem cell niche, and seed development. MIKE-MADS determines plant reproductive development (Teo et al., 2019). The bZIP TF is involved in plant developmental and physiological and stress responses (Corrêa et al., 2008).

The bHLH TFs are involved in plant growth and metabolism, photomorphogenesis, light signal transduction, and response to stresses (Sun et al., 2018). Katiyar et al. (2012) reported that MYB TFs play a key role in plant development, secondary metabolism, hormone signal transduction, and stress tolerance (Raza et al., 2022). The present findings are consistent with previous reports suggesting that *PeNAC* TFs may regulate plant growth, development, and stress responses while requiring further functional studies. The GO is mainly adapted to computational analysis and is used for the functionally annotate genes into distinct terms (Martin et al., 2004). KEGG resources become comprehensive knowledge for functional understanding and practical application of genomic information (Kanehisa et al., 2017). GO and KEGG annotation and enrichment analyses identified highly enriched terms in *PeNAC* genes (Figure 9) including transcription regulator activity, DNA binding, membrane-bounded organelle, organic substance biosynthetic process, primary metabolic and regulation of the biological process. The highly enriched KEGG pathways were associated with terpenoid-quinone biosynthesis, phosphatidylinositol signaling system and metabolism. These results are in consistent with previous reports on NAC by Gong et al. (2019), Yan H. et al. (2021), Mir et al. (2022).

Gene expression profiling provides important clues to determining the gene functions (Zhang et al., 2018). It has been reported that some NAC genes are specifically expressed in certain tissues and play crucial roles in plant growth and development (Shan et al., 2020). In the current study, diverse expression levels of *PeNAC* genes were found between passion fruit leaves, peels, pulps, and roots tissues (Figure 10). Among the expressed *PeNAC* genes, the highest 95 (90%) *PeNAC* genes were expressed in pulps followed by 84 (80%) *PeNAC* genes in leaves, 83 (79%) *PeNAC* genes in roots, and only 35 (33%) *PeNAC* genes were expressed in peels (Figure 10). Our results are consistent with Li et al. (2020), who also found the highest expressions of *NAC* genes in banana (*Musa acuminata*) pulp compared with other tissues. In addition, *PeNAC014*, *PeNAC062*, *PeNAC082*, and *PeNAC094* showed high expression levels in passion fruit peel and pulp. Similarly, *PeNAC010*, *PeNAC014*, *PeNAC039*, *PeNAC053*, *PeNAC075*, and *PeNAC100* had the highest expression levels in passion fruit leaves and roots. Zhang et al. (2018) also found that 13 of 128 *NAC* genes were differentially expressed at fruit growth and developmental stages in apple. Li X. et al. (2021) reported that out of 114 walnut *JmNAC* genes, 17 and 14 *JmNAC* genes were specifically expressed in walnut exocarp and embryo tissues.

It has been reported that NAC family members also play significant roles in plant responses to biotic and abiotic stresses (Tran et al., 2004; Huang et al., 2015; Shao et al., 2015; Hu et al., 2022). The possible role of *PeNAC* genes in passion fruit under biotic (*F. kyushuense*) and drought stress conditions were analyzed at qRT-PCR expression levels. Nearly, all the tested *PeNAC* genes were either positively or negatively regulated under both stress conditions (Figures 11, 12). Genes such as *PeNAC001, PeNAC003, PeNAC028, PeNAC033, PeNAC057, PeNAC058*, *PeNAC063*, and *PeNAC077* were highly upregulated (>5-fold) at 9 and 12 dpi in yellow and purple passion fruit under *F. kyushuense* stress (Figure 12). Moreover, under drought stress conditions, among tissues and cultivars, the significantly highest expression levels were found in Y-stem (*PeNAC041*, >64-fold, Figure 11G) followed by Y-root (*PeNAC088*, >40-fold, Figure 11N), P-leave (*PeNAC013*, >40-fold, Figure 11E) and Y-root (*PeNAC008* and *PeNAC033*, >11-fold, Figures 11C,F), whereas *PeNAC007* and *PeNAC011*were not expressed under *F. kyushuense* stress and drought stress conditions in both cultivars (Figure 11D), respectively.

Similar results were found by Yan H. et al. (2021) in sweet potato (*Ipomoea triloba* and *I. trifida*) *ItbNAC* and *ItfNAC* genes that showed diver expressions under drought stress conditions. Five genes including *ItbNAC110, ItbNAC114, ItfNAC15, ItfNAC28*, and *ItfNAC62* were highly upregulated (>20-fold) under drought stress. Tran et al. (2004) reported that the *ANAC019, ANAC055*, and *ANAC072* genes expression were upregulated in *Arabidopsis* and *SlNAC4* in tomato under drought stress (Zhu et al., 2014). Overexpression of *OsNAC22* and *OsNAC52* improved the drought and salt tolerance in rice (Gao et al., 2010; Hong et al., 2016), *MdNAC1* in apple (Jia et al., 2019). Wang et al. (2009) reported that *ATAF1* NAC TF negative regulated the defense responses against pathogens. *TaNAC4* gene is involved in wheat response to biotic and abiotic stresses (Xia et al., 2010).

## Conclusion

In this study, a comprehensive analysis identified 105 *PeNAC* genes in the passion fruit genome. The physicochemical properties, gene structures, evolution, and expression patterns of *PeNAC* genes were determined. All 105 *PeNAC* genes were phylogenetically divided into fifteen clades and intron-intron structure, conserved motifs, protein–protein interactions network and 3D structures were highly conserved suggesting their function conservation. Furthermore, syntenic analyses, TFs regulatory network analysis, GO, KEGG annotation, and putative miRNA prediction analysis were also performed. FPKM-based gene expression profiles of *PeNAC* genes exhibited a diverse expression in passion fruit root, peel, leaves, and pulp tissues. The qRT-PCR expression analysis suggested that most of the *PeNAC* genes were highly upregulated under *Fusarium kyushuense* and drought stress conditions compared to controls. These findings provide a foundation for future studies on the functions of *PeNAC* genes in passion fruit and other plants.

## Data availability statement

The original contributions presented in this study are included in the article/Supplementary material, further inquiries can be directed to the corresponding author.

## Author contributions

FC, QY, and HMR contributed to conceptualization and validation. QY, HMR, JZ, and MS contributed to methodology. QY, BL, and HMR contributed to software. QY, BL, and TG contributed to formal analysis. QY and HMR contributed to data curation, writing, and original draft preparation. FC and HMR contributed to review and editing. FC contributed to supervision and funding acquisition. All authors have read and agreed to the published version of the manuscript.

## Footnotes

1
https://www.arabidopsis.org/

2
http://planttfdb.gao-lab.org/family.php?sp=Ptr&fam=NAC

3
http://cucurbitgenomics.org/ftp/genome/cucumber/Chinese_long/v2/

4
https://www.ncbi.nlm.nih.gov/genome/?term=ziziphus+jujuba

5
http://plants.ensembl.org/Theobroma_cacao/Info/Strains?db=core

6
http://ftp.cngb.org/pub/CNSA/data3/CNP0001287/CNS0275691/CNA0017758/

7
https://github.com/CJ-Chen/TBtools/releases

8
https://pfam.xfam.org/

9
https://www.ncbi.nlm.nih.gov/Structure/bwrpsb/bwrpsb.cgi

10
http://smart.embl-heidelberg.de/

11
http://www.ebi.ac.uk/Tools/pfa/iprscan/

12
https://web.expasy.org/protparam/

13
http://www.csbio.sjtu.edu.cn/bioinf/plant-multi/

14
https://www.megasoftware.net/

15
https://itol.embl.de/

16
https://meme-suite.org/meme/tools/meme

17
http://bioinformatics.psb.ugent.be/webtools/plantcare/html/

18
https://string-db.org

19
http://www.sbg.bio.ic.ac.uk/phyre2/html/

20
https://www.zhaolab.org/psRNATarget/

21
https://cytoscape.org/

22
http://eggnog-mapper.embl.de/

23
http://plantregmap.gao-lab.org/binding_site_prediction.php

24
https://cytoscape.org/download.html

25
https://www.primer3plus.com/

26
https://www.graphpad.com/
