# Supplementary material for: Genome-wide identification and comprehensive analyses of NAC transcription factor gene family and expression analysis under Fusarium kyushuense and drought stress conditions in Passiflora edulis
Source: Front Plant Sci. 2022 Aug 25;13:972734. doi: 10.3389/fpls.2022.972734 (PMC9453495; doi:10.3389/fpls.2022.972734)
Supplement: Supplementary file 1 [file Data_Sheet_1.ZIP › Supplementary Material/Supplementary Table 3.docx]

**Supplementary Table 3.** Putative motifs identified from PeNAC proteins using MEME. The sequence logos were generated using WebLogo.

| **Motif name** | **Sequence logo** | **E-value** |
| --- | --- | --- |
| **Motif 1** | 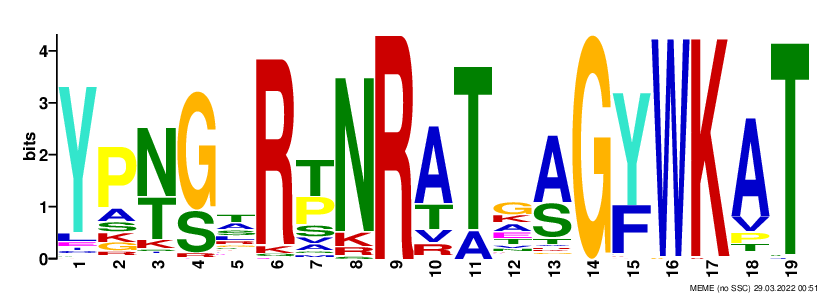  YPTGLRTNRATGAGYWKAT | 1.6e-1206 |
| **Motif 2** | 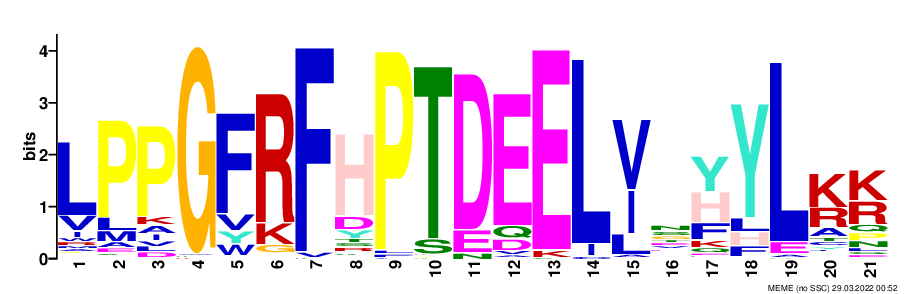  LPPGFRFHPTDEELVNYYLKK | 5.9e-1431 |
| **Motif 3** | 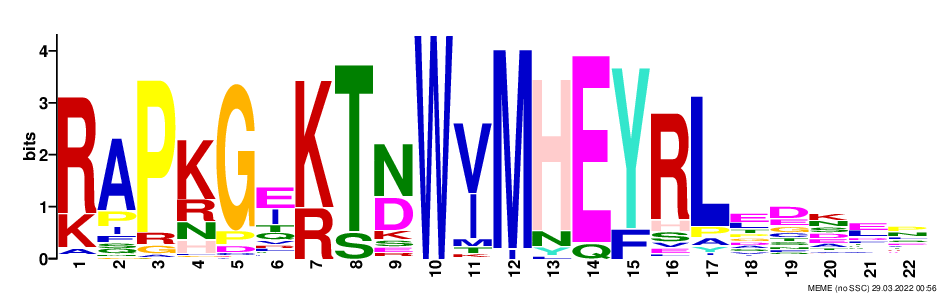  RAPKGEKTBWVMHEYRLEDKEP | 7.8e-1189 |
| **Motif 4** | 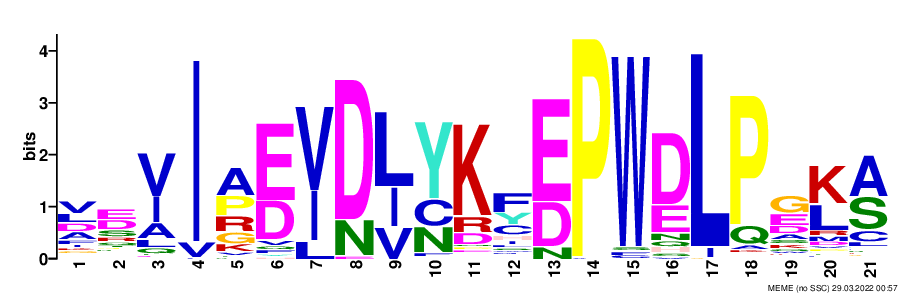  VEVIAEVDJYKFEPWDLPGKA | 3.2e-1028 |
| **Motif 5** | 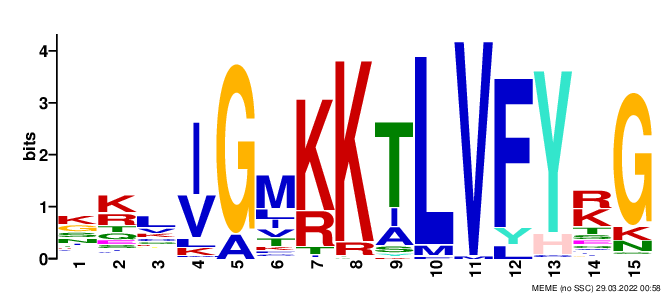  KKLIGMKKTLVFYRG | 2.3e-804 |
| **Motif 6** | 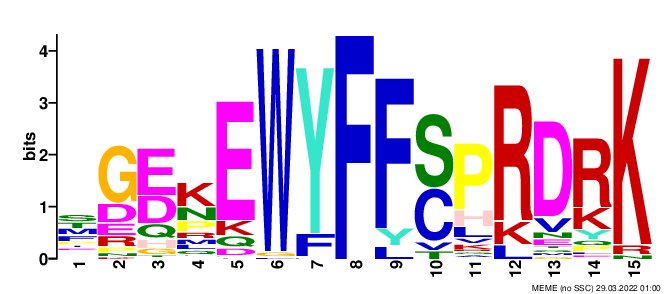  SGEKEWYFFSPRDRK | 1.7e-648 |
| **Motif 7** | 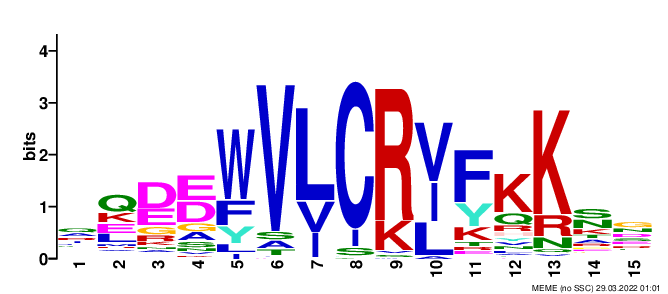  QQDEWVLCRVFKKSG | 2.4e-462 |
| **Motif 8** | 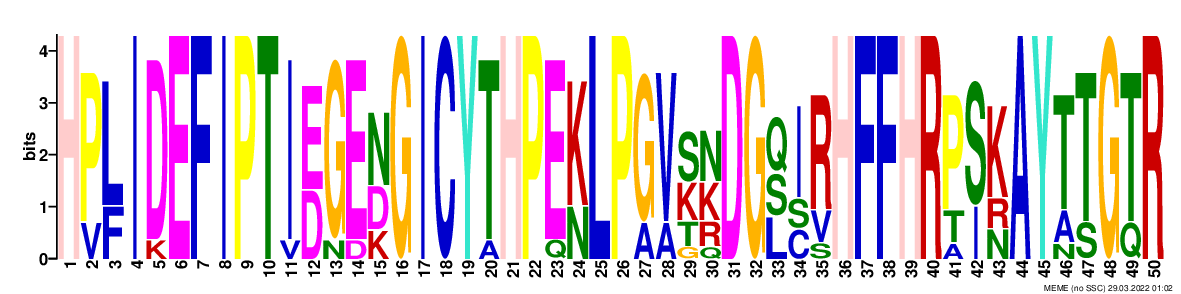  HPLIDEFIPTIEGEBGICYTHPEKLPGVSNDGQIRHFFHRPSKAYTTGTR | 7.4e-333 |
| **Motif 9** | 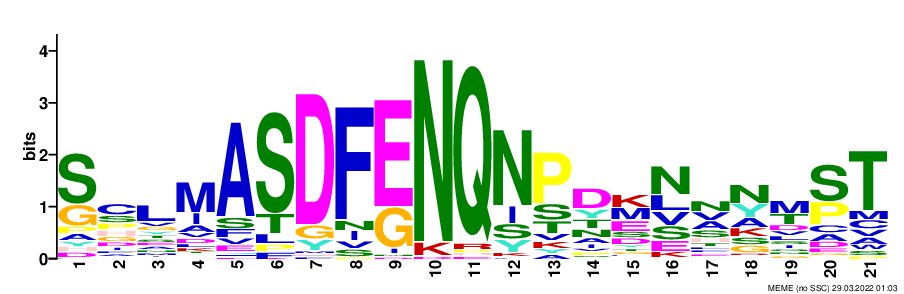  SCLMASDFENQNPDKNNNMST | 3.9e-325 |
| **Motif 10** | 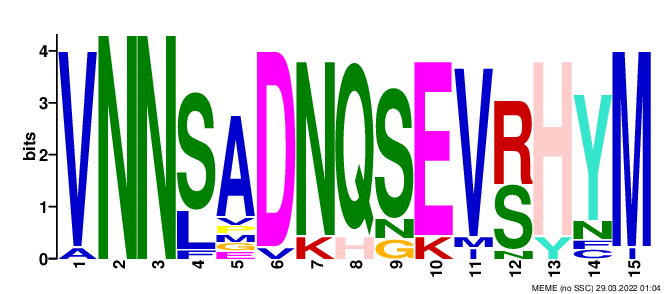  VNNSADNQSEVRHYM | 4.1e-139 |
